# An archaeal transcription factor EnfR with a novel ‘eighth note’ fold controls hydrogen production of a hyperthermophilic archaeon *Thermococcus onnurineus* NA1

**DOI:** 10.1093/nar/gkad699

**Published:** 2023-08-31

**Authors:** Da-Woon Bae, Seong Hyuk Lee, Ji Hye Park, Se-Young Son, Yuxi Lin, Jung Hyen Lee, Bo-Ram Jang, Kyu-Ho Lee, Young-Ho Lee, Hyun Sook Lee, Sung Gyun Kang, Byoung Sik Kim, Sun-Shin Cha

**Affiliations:** Department of Chemistry & Nanoscience, Ewha Womans University, Seoul 03760, Republic of Korea; Marine Biotechnology Research Center, Korea Institute of Ocean Science and Technology, Busan, South Korea; Department of Food Science and Biotechnology, Ewha Womans University, Seoul 03760, Republic of Korea; Department of Chemistry & Nanoscience, Ewha Womans University, Seoul 03760, Republic of Korea; Research Center for Bioconvergence Analysis, Korea Basic Science Institute (KBSI), Cheongju, Chungbuk 28119, Republic of Korea; Department of Food Science and Biotechnology, Ewha Womans University, Seoul 03760, Republic of Korea; Department of Life Science, Sogang University, 35 Baekbeom-Ro, Mapo-Gu, Seoul, South Korea; Department of Life Science, Sogang University, 35 Baekbeom-Ro, Mapo-Gu, Seoul, South Korea; Research Center for Bioconvergence Analysis, Korea Basic Science Institute (KBSI), Cheongju, Chungbuk 28119, Republic of Korea; Bio-Analytical Science, University of Science and Technology, Daejeon 34113, Republic of Korea; Department of Systems Biotechnology, Chung-Ang University, Anseong, Gyeonggi 17546, Republic of Korea; Frontier Research Institute for Interdisciplinary Sciences, Tohoku University, Sendai, Miyagi 980-8578, Japan; Marine Biotechnology Research Center, Korea Institute of Ocean Science and Technology, Busan, South Korea; Department of Marine Biotechnology, KIOST School, University of Science and Technology, Daejeon, South Korea; Marine Biotechnology Research Center, Korea Institute of Ocean Science and Technology, Busan, South Korea; Department of Marine Biotechnology, KIOST School, University of Science and Technology, Daejeon, South Korea; Department of Food Science and Biotechnology, Ewha Womans University, Seoul 03760, Republic of Korea; Department of Chemistry & Nanoscience, Ewha Womans University, Seoul 03760, Republic of Korea

## Abstract

*Thermococcus onnurineus* NA1, a hyperthermophilic carboxydotrophic archaeon, produces H_2_ through CO oxidation catalyzed by proteins encoded in a carbon monoxide dehydrogenase (CODH) gene cluster. TON_1525 with a DNA-binding helix-turn-helix (HTH) motif is a putative repressor regulating the transcriptional expression of the *codh* gene cluster. The T55I mutation in TON_1525 led to enhanced H_2_ production accompanied by the increased expression of genes in the *codh* cluster. Here, TON_1525 was demonstrated to be a dimer. Monomeric TON_1525 adopts a novel ‘eighth note’ symbol-like fold (referred to as ‘eighth note’ fold regulator, EnfR), and the dimerization mode of EnfR is unique in that it has no resemblance to structures in the Protein Data Bank. According to footprinting and gel shift assays, dimeric EnfR binds to a 36-bp pseudo-palindromic inverted repeat in the promoter region of the *codh* gene cluster, which is supported by an *in silico* EnfR/DNA complex model and mutational studies revealing the implication of N-terminal loops as well as HTH motifs in DNA recognition. The DNA-binding affinity of the T55I mutant was lowered by ∼15-fold, for which the conformational change of N-terminal loops is responsible. In addition, transcriptome analysis suggested that EnfR could regulate diverse metabolic processes besides H_2_ production.

## Graphical Abstract

**Graphical Abstract:**
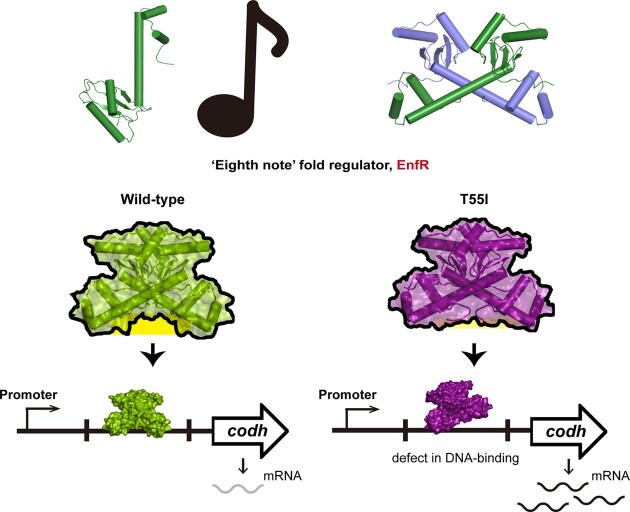

## INTRODUCTION

Fossil fuels such as coal, oil and natural gas have been major energy sources responsible for almost 80% of a total energy supply for decades (1,2). However, due to air pollution and global warming caused by their combustion, fossil fuel consumption is declining at a global level and the demand for alternative renewable energy sources is soaring (3). According to a roadmap to 2050 reported by the International Renewable Energy Agency (IRENA), the percentage share of renewable energy would be reached 65% in 2050, whereas the use of fossil fuels would fall to one-third of the present levels (4). Hydrogen is an attractive candidate as a future fuel for several reasons. It is a non-toxic, clean and efficient source in that the major byproduct from hydrogen combustion is pure water, and its energy content per unit mass is 3-times higher than traditional fossil fuels (5). Therefore, the global demand for hydrogen energy is getting higher, and the development of hydrogen-fueling infrastructures for production, storage and utilization is a great research challenge (6–8).

Almost 96% of hydrogen is produced using natural gas (48%), petroleum (30%) and coal (18%) (9). Besides H_2_ production depending on nonrenewable fossil sources, there are ways to produce H_2_ from renewable feedstocks such as wind, solar and biomass (10). Microbial H_2_ production is also promising since it is an environment-friendly and less energy-intensive way to produce H_2_. It is also notable that microbes can exploit industrial wastes including carbon monoxide (CO) or organic matter to produce H_2_ (11–13). In particular, biohydrogen production using thermophiles at high temperatures (≥60°C) has numerous advantages such as high metabolic activity leading to enhanced product formation rates (14), less contamination by H_2_-consuming microorganisms (15), no intensive cooling necessary during fermentation, decreased density, surface tension, and viscosity of culture broth, etc. (16). *Thermococcales*, *Thermotogales*, *Desulfurococcales* and *Clostridium* species have been reported to produce H_2_ from organic substrates at high temperatures (17–19).

*Thermococcus onnurineus* NA1, isolated from a deep-sea hydrothermal vent, is a sulfur-reducing hyperthermophilic carboxydotrophic archaeon (20). It requires elemental sulfur as a terminal electron acceptor for heterotrophic growth on peptides or an amino acid mixture and exhibits optimum growth at 80°C and pH 8.5. Interestingly, *T. onnurineus* NA1 grows under diverse substrates including CO and formate to produce H_2_ (21). The H_2_ productivity of *T. onnurineus* NA1 is higher than that of other hyperthermophilic archaea such as *Pyrococcus furiosus* and *Desulfurococcus amylolyticus* (18,21,22). In the case of the CO-utilization, the unique *codh* gene cluster in *T. onnurineus* NA1, which encodes CO dehydrogenase, hydrogenase and Na^+^/H^+^ antiporter, catalyzes the anaerobic oxidation of CO to CO_2_ through the water-gas shift reaction: CO + H_2_O → H_2_ + CO_2_ (Δ*G*°′ = −20 kJ/mol) (23).

In a previous study, an adaptive evolution was carried out through the serial transfer of *T. onnurineus* NA1 into CO medium to enhance its H_2_ production (24). After the 156-time serial transfer, cell density, CO consumption rate, and H_2_ production rate increased by 2.8-, 6.5- and 5.9-fold, respectively. Although mutations occurred in gene sequences of ten proteins after the serial transfer (24), the CO consumption rate and H_2_ production rate of a mutant strain MC11 harboring the Thr → Ile replacement at position 55 in the TON_1525 protein were comparable to those of the 156-serial-transferred strain (156T). Consistently, the expression of proteins essential for CO-dependent H_2_ production was increased in both 156T and MC11 strains compared to the wild-type (24).

Sequence analysis revealed that TON_1525 is a Tfx DNA-binding protein. According to NCBI’s Gene resources, a total of 288 microorganisms have Tfx DNA-binding proteins, most of which (∼98%) are distributed in archaea, including *Thermococcales*, *Methanococcales*, and *Halobacteriales*. Almost all archaeal Tfx DNA-binding proteins have a basic DNA-binding helix-turn-helix (HTH) motif (p*I* range = 8.8–11.0) at their N-terminal region and an acidic C-terminal domain (pI range = 4.6–6.8) in common. The amino acid sequence of the HTH motif resembles that of the bacterial RNA polymerase (RNAP) σ^70^ region4 domain that recognizes specific transcription-initiation sites in the promoter region and recruits RNAP to start transcription (25). The presence of the DNA-recognizing HTH motif, together with experimental data that two Tfx family members (TON_1525 and the MTH0916 protein from *Methanobacterium thermoautotrophicum*) bind to promoter regions (24,26), strongly suggests that the Tfx family members are transcription regulators. Nevertheless, there is no report about the basic characteristics of Tfx family members such as the molecular shape, the oligomerization state, the target DNA sequence, and the DNA-recognition mode. In this study, we revealed that TON_1525 adopts a fold resembling an eighth note symbol and the unique X-shaped dimeric structure of EnfR has no structural homolog. In addition, the target DNA sequence was determined in the promoter region of the *codh* gene cluster, and the DNA-binding mode of EnfR was elucidated based on structural and mutational analyses. We also provide a possibility that it may be involved in the transcriptional regulation of diverse metabolic processes in *T. onnurineus* NA1. Consequently, our study will play as a platform for future research about the Tfx family members and for the understanding of how the energy metabolism of a hyperthermophilic archaeon *T. onnurineus* NA1 is controlled at the transcription level.

## MATERIALS AND METHODS

### Protein preparation

The *T. onnurineus* NA1 TON_1525 gene encoding residues 1–146 was synthesized to have the in-frame non-cleavable C-terminal His6-tag and inserted at the *Nde*I and *Not*I sites of the expression vector pET24a(+) (Novagen, USA). The T55I substitution was generated by site-directed mutagenesis using the wild-type gene as a template. The wild-type and mutant construct were transformed into *Escherichia coli* Rosetta (DE3), respectively. The two transformed cells were grown to an optical density values at 600 nm (OD_600_) of ∼0.6 in Luria-Bertani medium at 37°C, and the expression of the wild-type and mutants were induced by 0.5 mM isopropyl β-d-thiogalactopyranoside. After 4 h induction at 37°C, the cells were harvested, resuspended in a 10 mM Tris (pH 7.4), 500 mM NaCl and 5 mM β-mercaptoethanol buffer, and disrupted by sonication. The crude lysate was centrifugated at 10,000 × *g* for 30 min and the resulting supernatant was then boiled at 75°C for 10 min. The lysate was centrifugated again at 10,000 × *g* for 1 h. The resulting supernatant was loaded onto a nickel-nitrilotriacetic acid (Ni-NTA) column (GE Healthcare, USA). The protein fraction eluted from the Ni-NTA column was concentrated and loaded onto a Superdex 75 HR 16/600 column (GE Healthcare, USA). The elute from gel-filtration was concentrated to ∼10 mg/ml in a 20 mM Tris buffer (pH 7.4), 500 mM NaCl and 2 mM DTT for crystallization.

### Crystallization, X-ray diffraction experiment and structure determination

The microbatch crystallization method was employed to grow crystals under oil (27). Crystals of the wild-type TON_1525 were obtained at 22°C by mixing 1 μl of protein solution with an equivalent volume of a mother liquor consisting of 30% polyethylene glycol (PEG) 400, 100 mM HEPES (pH 7.5), and 200 mM NaCl. The T55I mutant was crystallized in the same way with a mother liquor of 30% PEG 400, 100 mM HEPES (pH 7.5), and 200 mM ammonium sulfate. A 2.8 Å resolution data set of the wild-type and a 3.4 Å resolution data set of the T55I mutant were collected at beamline 17A of Photon Factory, Tsukuba, Japan. Both data sets were integrated and scaled with *XDS* (28). Crystals of the wild-type belonged to the space group *P*4_3_2_1_2 with cell parameters *a* = *b* = 102.7 Å and *c* = 90.4 Å corresponding to two monomers in an asymmetric unit. Crystals of the T55I mutant, which belonged to the space group *P*4_3_2_1_2 with cell parameters *a* = *b* = 123.2 Å and *c* = 82.7 Å, contain two monomers in an asymmetric unit (Supplementary Table S1).

To solve the structures of the wild-type and the T55I mutant by molecular replacement (MR), we used the MTH0916 structure (PDB code:1NR3) as a search model because MTH0916 is the only Tfx family member whose structure is available. However, our MR trials using programs *MOLREP* and *PHASER* were unsuccessful. TON_1525 has no methionine residue except for the N-terminal initiation methionine that is usually disordered in proteins, which indicates that TON_1525 is not adequate for incorporating seleno-methionines for *de novo* phasing. Therefore, a leucine residue at position 83 was replaced by methionine together with another C135S substitution in the T55I mutant. Since a protein band corresponding to the dimeric size of TON_1525 was observed on non-reducing SDS-PAGE during protein purification, we mutated Cys135 to serine to prevent intermolecular disulfide bonds. After the introduction of the Cys → Ser replacement, the band of the dimeric size disappeared. For the seleno-methionine labeling of the triple mutant (T55I/L83M/C135S), the methionine auxotroph *E. coli* B834 (DE3) (Novagen) strain was used as a host. The triple mutant harboring a seleno-methionine at position 83 was purified in the same way as the wild-type and was concentrated to ∼10 mg/ml for crystallization.

Crystals of the triple mutant were obtained in the same way as the wild-type with a mother liquor consisting of 16% PEG 400, 100 mM Tris (pH 8.6) and 200 mM ammonium sulfate. A single-wavelength (0.9796Å) anomalous diffraction (SAD) data set was collected to the resolution of 3.00 Å at beamline 5C of Pohang Accelerator Laboratory and was processed by *HKL2000* (29). The experimental SAD phasing and the initial model building were performed with *PHENIX* (30). The initial model of the triple mutant was subjected to the automated model building of *BUCCANEER* (31) and then further refined to *R*/*R*_free_ of 0.251/0.267 by using *COOT* (32) and *PHENIX*. The crystal structures of the wild-type and the T55I mutant were determined with the triple mutant structure as a search model. MR solutions from *PHASER* was manually manipulated by *COOT* and refined by *PHENIX*. Several rounds of refinements and manual refitting gave rise to final models of the wild-type (*R*/*R*_free_ = 0.222/0.244) and the T55I mutant (*R*/*R*_free_ = 0.236/0.275). The final model of the wild-type contains residues 4∼142 of chain A and residues 4∼139 of chain B while that of the T55I mutant consists of residues 4–144 of chain A and residues 2–139 of chain B. The Ramachandran plots indicate 97.1% (the wild-type) and 91.6% (the T55I mutant) of non-glycine residues are in the most favored regions, and all others are in the additionally allowed regions.

### Analytical ultracentrifugation

Sedimentation velocity of wild-type EnfR and all the mutants were calculated by analytical ultracentrifugation at a wavelength of 280 nm using a ProteomeLab XL-A centrifuge (Beckman Coulter, Brea, CA, USA) equipped with an AN-60 Ti rotor at 20°C. Data from sedimentation at 42,000 rpm were collected at 5-min intervals, giving a total of 130 scans. Sedimentation velocity data were analyzed using *SEDFIT*. The density and viscosity of the buffer solution were determined at 20°C to be 1.019 g/ml and 0.010503 P, respectively. The partial specific volume was calculated as 0.7454–0.7466 ml/g at 20°C.

### DNase I protection assay

For DNase I protection assay, a DNA probe of the 150-bp promoter region of the *codh* gene cluster (24) was amplified by PCR using 6-carboxyfluorescein (6-FAM)-labeled 1017_150_F and unlabeled 1017_150_R as primers (Supplementary Table S2). The labeled DNA probe (200 ng) was then incubated with the purified EnfR protein for 10 min at 80°C in a 20 μl reaction mixture containing 1 × binding buffer (20 mM Tris–HCl pH 7.5, 200 mM KCl, 1 mM EDTA, and 5% glycerol). DNaseI digestion of the protein-DNA complex followed the procedures described previously (33). The resulting DNA fragments were precipitated with ethanol, eluted in nuclease-free water and then analyzed using an ABI 3730xl DNA analyzer (Applied Biosystems, Foster City, CA) with Peak Scanner™ Software ver. 1.0 (Applied Biosystems).

### Molecular docking

An *in silico* model of EnfR in complex with the 36-bp target DNA was built using the *HADDOCK* v.2.4 server. For molecular docking, we defined the α2-turn-α3 region (residues 24–51) of EnfR as active residues that were predicted to be involved in DNA-binding, and the first three N-terminal residues that are conformationally different from that of the T55I mutant structure were also selected as active residues in each monomer. For DNA molecule, active residues were selected based on the sequence of 5′-AATCTTTTTGTTTACATT-3′ which was determined as a minimal binding region in the DNase I protection assay. Passive residues were automatically selected by the server. Among the generated models, the top-ranked model was selected to get insights into the DNA-binding mode of EnfR.

### Electrophoretic mobility shift assay (EMSA)

All protein mutants were generated by site-directed mutagenesis using the wild-type TON_1525 (EnfR) gene as a template (Cosmogenetech, Republic of Korea). Mutant proteins were prepared by the same procedure as described in *Protein Preparation*. To analyze interactions between EnfR and the target DNA sequence, the 36-bp 5′-Cyanine 5 (Cy5)-labeled (5 nM) probes were used with different protein concentrations. For verification of EnfR-binding to the promoter regions of differentially expressed genes (DEGs), DNA probes (10 nM) of TON_1582, TON_0537 and TON_1563 genes were amplified by PCR using 5′-Cy5-labeled forward primers (1582_F, 0537/8_F and 1563_F) and unlabeled reverse primers (1582_R, 0537/8_R and 1563_R) (Supplementary Table S4). Proteins were incubated with the probes at 37°C for 20 min in a buffer containing 10 mM Tris (pH 7.4), 200 mM KCl, 2 mM DTT, 0.2 mM EDTA. After incubation, the reaction mixtures were separated by electrophoresis using a 6% polyacrylamide gel. The gels were analyzed with ChemiDoc MP imaging system (Bio-Rad). The band intensities were calculated with the program *ImageJ* and used to compare the DNA-binding affinities of the wild-type and the T55I mutant.

### Strains, culture conditions and analytical methods

*T. onnurineus* NA1 (KCTC 10859) was routinely cultured in modified medium 1 (MM1) supplemented with 1 bar of 100% CO (MM1 + CO) in serum bottles under anaerobic conditions at 80°C (34). An MC11 mutant strain harboring the amino acid substitution, T55I, in EnfR was previously constructed (24) and used in this study. For the cultivation of the wild-type and MC11 strains in the bioreactor, 100% CO was supplemented at a flow rate of 120 ml/min and the pH was controlled at 6.1–6.2 using 5 N NaOH containing 3.5% NaCl. Cell growth was monitored by OD_600_, and H_2_ production was analyzed as previously described (35).

### Transcriptome analysis

The wild-type and MC11 strains were grown to the exponential growth phase in MM1 + CO medium and three independent cultures were employed for RNA extraction. Total RNA was extracted from the cells using Trizol reagent (Invitrogen, Carlsbad, CA) as previously described (24). The quality and quantity of the RNAs were assessed as RNA integrity number (RIN) (36) and RNA electropherograms produced with an Agilent 2100 Bioanalyzer (Palo Alto, USA) according to the manufacturer's instructions. 10 μg of RNA from samples with RIN values ranging from 8.4 to 9.7 for the wild-type and MC11 strains was used for further experiments. Sequencing library generation and sequencing using a HiSeq 2500 (Illumina, San Diego, USA) were performed by CJ bioscience (Seoul, Republic of Korea). Quality-filtered reads were mapped against a reference genome sequence (BioProject accession number PRJNA59043) (https://www.ncbi.nlm.nih.gov/bioproject) by Gibiome (Seongnam, Republic of Korea). Relative transcript abundance was measured as fragments per kilobase per million mapped reads (FPKM) (37). RNA sequence data were submitted to the NCBI Gene Expression Omnibus (GEO) database (http://www.ncbi.nlm.nih.gov/geo) with accession code GSE200806.

### Western blotting analysis

After immunization of rabbits with each purified protein in Ab Frontier (Seoul, Republic of Korea), polyclonal antibodies were generated and verified by Western blotting. Western blotting analysis was conducted as described (13).

### Isothermal titration calorimetry (ITC)

ITC experiments were conducted using a VP-ITC instrument from Malvern Panalytical (Malvern, UK) at 25°C. The wild-type, the T55I mutant, and the 36-bp target DNA were dissolved in 10 mM Tris–HCl buffer (pH 7.4) containing 200 mM KCl, 1 mM tris(2-chloroethyl) phosphate (TCEP) and 0.2 mM EDTA. The solutions were degassed for 3 min before being loaded into the ITC instrument. The concentration of the wild-type and the T55I mutant in the cell was 25 μM, while the concentration of the 36-bp target DNA in the syringe was 380 μM. Titration experiments consisting of 25 injections in total were performed with continuous stirring at 307 rpm. The injection volume was 2 μl for the first injection to minimize effects of bubbles and 11 μl for the remaining injections. The initial delay and the reference power were set to 1200 s and 10 μcal/s, respectively. The 36-bp target DNA solution was titrated into the same buffered solution for the heat of dilution. Binding isotherms were displayed after subtracting the dilution heat and baseline correction. Fitting analyses of binding isotherms were carried out using the one-set-of-sites binding model in the MicroCal Origin 7.0 software.

### Luciferase reporter assay

A luciferase reporter assay to monitor the specific binding of EnfR to the target DNA sequence was designed by using a heterologous bacterial host *E. coli* (38). To construct the EnfR-producing plasmids, wild-type and mutant TON_1525 (EnfR) genes in the expression plasmids were subcloned into pJK1113, a plasmid containing the arabinose-inducible promoter P_BAD_ (Supplementary Table S5) (39). The resulting plasmids were designated as the pJH_EnfR series. To construct synthetic promoters that are repressed by EnfR, we engineered two constitutively expressing bacterial promoters (J23117 and J23110) (40) to have the EnfR-binding site either at the very downstream of or at partially overlapping position with the -10 box for bacterial RNAP (see ‘The target DNA sequence and the implication of the N-terminal loop in DNA recognization’ of the Results and Discussion section). DNA fragments containing such engineered sequences (Supplementary Table S6) were chemically synthesized and then cloned into the plasmid pBBR_lux containing a promoterless *luxCDABE* operon (Supplementary Table S5) (41). The resulting reporter plasmids were designated as the pJH_road-blocking (if EnfR binds to the downstream of -10 box) or pJH_steric_ hindrance (if EnfR binds to the -10 box thereby interfering RNAP binding). Finally, to produce reporter strains, each pJH_EnfR plasmid was co-transformed with pJH_road-blocking or pJH_steric_hindrance into *E. coli* DHα cells (Supplementary Table S5).

For the luciferase reporter assay, overnight cultures of the reporter strains were inoculated into the fresh LB medium supplemented with appropriate antibiotics and L-arabinose for EnfR induction. When the cells were grown to an exponential phase (OD_600_ of 0.5), 200 μl of the culture was transferred into a well of the Nunc^TM^ 96-well white/clear bottom plate (Thermo Fisher Scientific, MA). After further incubation at 37°C for 3 h, the OD_600_ and cellular luminescence were measured using the Spark™ microplate reader (Tecan, Switzerland). The relative luminescence unit (RLU) was derived by dividing the cellular luminescence with OD_600_. The relative luminescence level was then calculated by normalizing the RLU of each sample with that of the EnfR-uninduced sample [Relative luminescence level = (RLU of the sample / RLU of the EnfR-uninduced sample) × 100 (%)].

## RESULTS AND DISCUSSION

### TON_1525 is a homo-dimer

**Figure 1.**
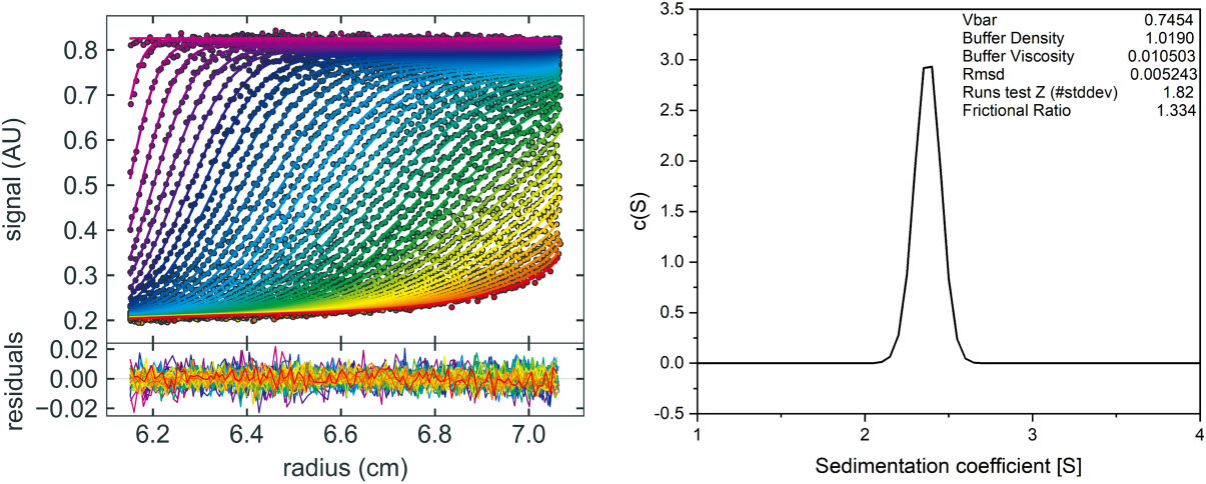
Determination of the oligomeric state of EnfR (TON_1525). Sedimentation Velocity Ultracentrifugation Analysis (SV-AUC) of EnfR. *Left panel*: raw sediment velocity profiles recorded using absorbance at 280 nm. *Right panel*: the distribution of sedimentation coefficient *c*(s) from the model. The analytical ultracentrifugation profile of EnfR showed that it exists as a dimer in solution.

Proteins engaged in transcriptional regulations are generally homo-oligomers, and thus, we examined the oligomeric state of TON_1525 through the sedimentation velocity analytical ultracentrifugation (SV-AUC) (Figure 1). Occupying 99.98% of the total protein peak area, it was analyzed to have a molecular weight of 33,800 Da. Consequently, it can be safely concluded that TON_1525 exists as a homo-dimer in solution.

### TON_1525 adopts an ‘eighth note’ symbol-like fold that is assembled into an X-shaped dimer

**Figure 2.**
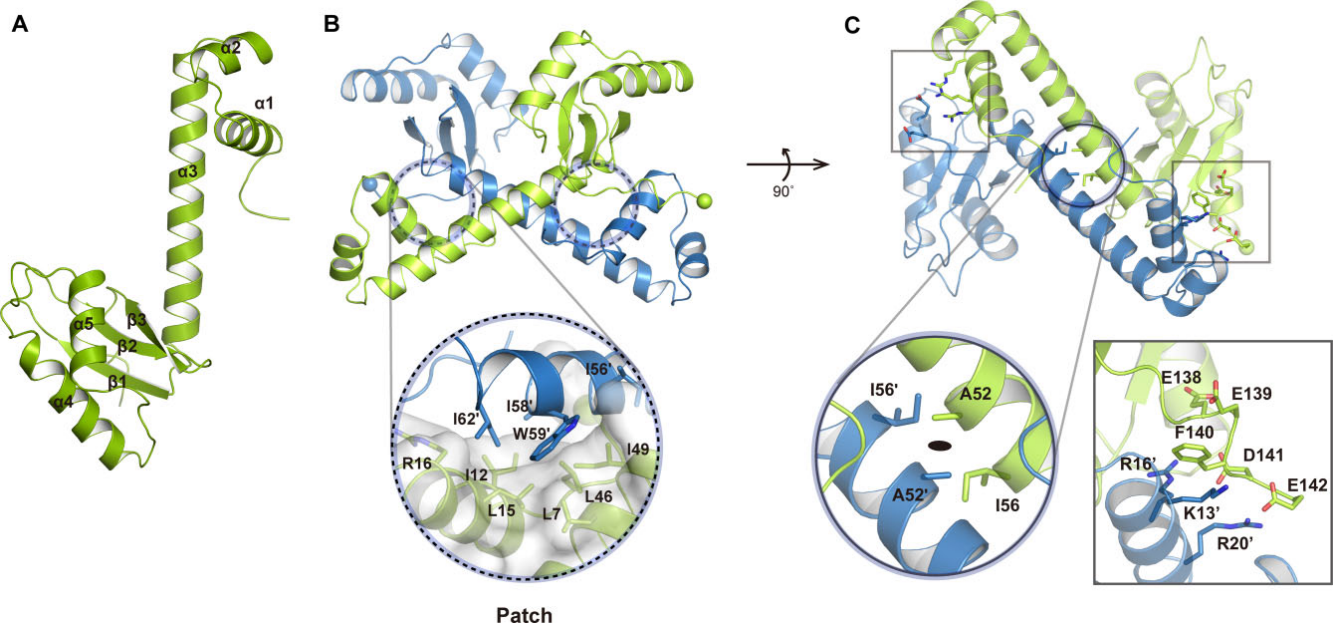
The overall structure of monomer, dimer and the dimeric interface of EnfR. (**A**) Monomeric structure of EnfR. (**B**) Side view of dimeric EnfR. Hydrophobic interactions between monomers are indicated by black dashed circles, and the hydrophobic patch is presented by white surface. (**C**) Bottom view of dimeric EnfR. Electrostatic interactions between the acidic EEFDE segment at the C-terminal region of one monomer and basic residues from the other monomer are indicated by two black squares. The black circle in the dimeric center represents a hydrophobic core. The molecular 2-fold axis is represented by a black oval at the central intersection. Each monomer is colored light green and blue, respectively. All spheres indicate the N- and C-termini of monomers. All residues participating in dimeric interactions are in sticks. For brevity, close-up views are shown only for one monomer in (B) and (C).

We determined the 2.8 Å resolution crystal structure of the wild-type TON_1525 (Supplementary Table S1). There are two monomers in the asymmetric unit of the wild-type TON_1525 crystals, which is compatible with our demonstration that TON_1525 is a dimeric protein in solution. The structures of the two monomers are virtually identical; their structures are superposed with the root mean square (r.m.s.) deviation of 0.490 Å for all Cα atoms. Monomeric structure of TON_1525 resembles an ‘eighth note (♩)’ symbol in that it can be divided into three parts: a flag, a straight stem and a note-head (Figure 2A) (hereafter referred to as ‘eighth note’ fold regulator, EnfR). The flag part contains two N-terminal α1 and α2 helices (residues 1–34), the stem part is a long α3 helix (residues 35–62) and the note-head corresponds to the C-terminal α+β domain (residues 63–146) in which a three-stranded antiparallel β-sheet (↑β1-↓β2-↑β3) is sandwiched by the stem helix (α3) on one face and two C-terminal helices (α4 and α5) on the other face (Figure 2A). The C-terminal α+β note-head domain has a β_A_-α_A_-α_B_-β_B_-β_C_ topology (the alphabet subscripts indicate the order of secondary structural elements represented by Greek letters) (Supplementary Figure S1). This domain is unique in that most proteins having β-α-α-β-β topology among the proteins belonging to the α+β class defined by Structural Classification of Proteins (SCOP) (42) shows β_A_-α_A_-α_B_-β_C_-β_B_ topology, which displayed a reversed order of the second and third β-strands compared with EnfR.

The dimerization of EnfR is mediated mainly by the flag and stem helices with a contact area of ∼2300 Å^2^, covering one-third of the molecular surface of a monomer. The long stem helices from two monomers form antiparallel X-shaped cross interactions (Figure 2B). Ala52 and Ile56 from a monomer form a hydrophobic core with the same residues from another monomer at the central intersection where the molecular 2-fold axis is located (Figure 2C). Three hydrophobic residues (Ile58, Trp59, and Ile62) at one end of the stem helix in a monomer make extensive contacts with a hydrophobic patch lined by Leu7, Ile12, Leu15, Arg16 (a three-carbon aliphatic chain in its side chain), Leu46, and Ile49 at the peripheral of the core (Figure 2B). To verify the importance of the hydrophobic interactions for the dimeric conformation, an EnfR mutant harboring three point mutations (A52W, I58G and I62G) was designed. The introduction of Trp into position 52 at the hydrophobic core was for steric clashes, and the two Ile → Gly mutations were to reduce contacts with the hydrophobic patch. As expected, SV-AUC revealed that 72.3% of the total protein peak area was estimated to have a monomeric size (Supplementary Figure S2). In addition to hydrophobic dimeric contacts, electrostatic interactions contribute in part to the dimerization. The acidic EEFDE segment (residues 138–142) of the note-head α+β domain in one monomer makes interactions with a basic side of α1 harboring Lys13, Arg16, and Arg20 in the other monomer (Figure 2C).

*Mycobacterium tuberculosis* RslA-bound RNA polymerase sigma factor σ_4_^L^ is the highest structural homolog of monomeric EnfR searched by the *Dali* server (Supplementary Table S7) (43). However, σ_4_^L^ displays a low structural similarity only towards the α2-turn-α3 HTH motif of EnfR (*Dali Z*-score of 8.5, r.m.s. deviation of 1.8 Å) (Supplementary Figure S3 and Table S7). In addition, the dimerization mode of EnfR is not observed among all structural homologs with *Dali Z*-score >6. The unique tertiary and quaternary structures of EnfR resembling the eighth-note indicate clearly that the Tfx family is structurally distinct from other transcription factor families.

In addition to DNA-binding domains (DBD), transcription factors generally have extra domains that mediate oligomerization and/or recognize molecular signals such as small molecules or proteins. For example, TrmB from *Thermococcales* and *Halobacterium salinarum* contains an N-terminal DBD and an effector binding domain (EBD). Sugar such as sucrose and maltotriose binds to EBD and induces a conformational change of TrmB to control the transcription of genes related to sugar metabolism (44,45). In EnfR, the C-terminal α+β note-head domain corresponds to the extra domain. As an effort to get an idea of the functional role of the note-head domain, we searched for structural homologs using the *Dali* server (Supplementary Table S8). A part of the *E.coli* 4-amino-4-deoxychorismate lyase (PDB code: 1I2L) was identified as the highest structural homolog; the *Z*-score was 6.1, and the r.m.s. deviation was 3.2 Å for 73 matching C_α_ atoms. Structural investigation revealed that the homologous region in the lyase is implicated in dimerization, which suggests that the note-head domain of EnfR appears not to function as an EBD.

The absence of EBD, which senses molecular signals and induces structural alteration, suggests that an antirepressor/repressor system (*Ant*/*Rep*) might regulate the DNA-binding activity of EnfR. *Ant* is generally known to bind to DNA-recognition sites of *Rep* to prevent the formation of the *Rep*-operator complex (46). There are also novel types of *Ant* that bind not to DNA-binding sites but to other regions to inactivate *Rep* (47). In these ways, *Ant* disrupts the *Rep*-operator complex, allowing the transcriptional machinery to initiate gene expression. Unfortunately, there is no known *Ant* of EnfR. Consequently, it is a future challenge to reveal how the negative regulation of the *codh* gene cluster by EnfR is relieved.

### The target DNA sequence and the implication of the N-terminal loop in DNA recognization

**Figure 3.**
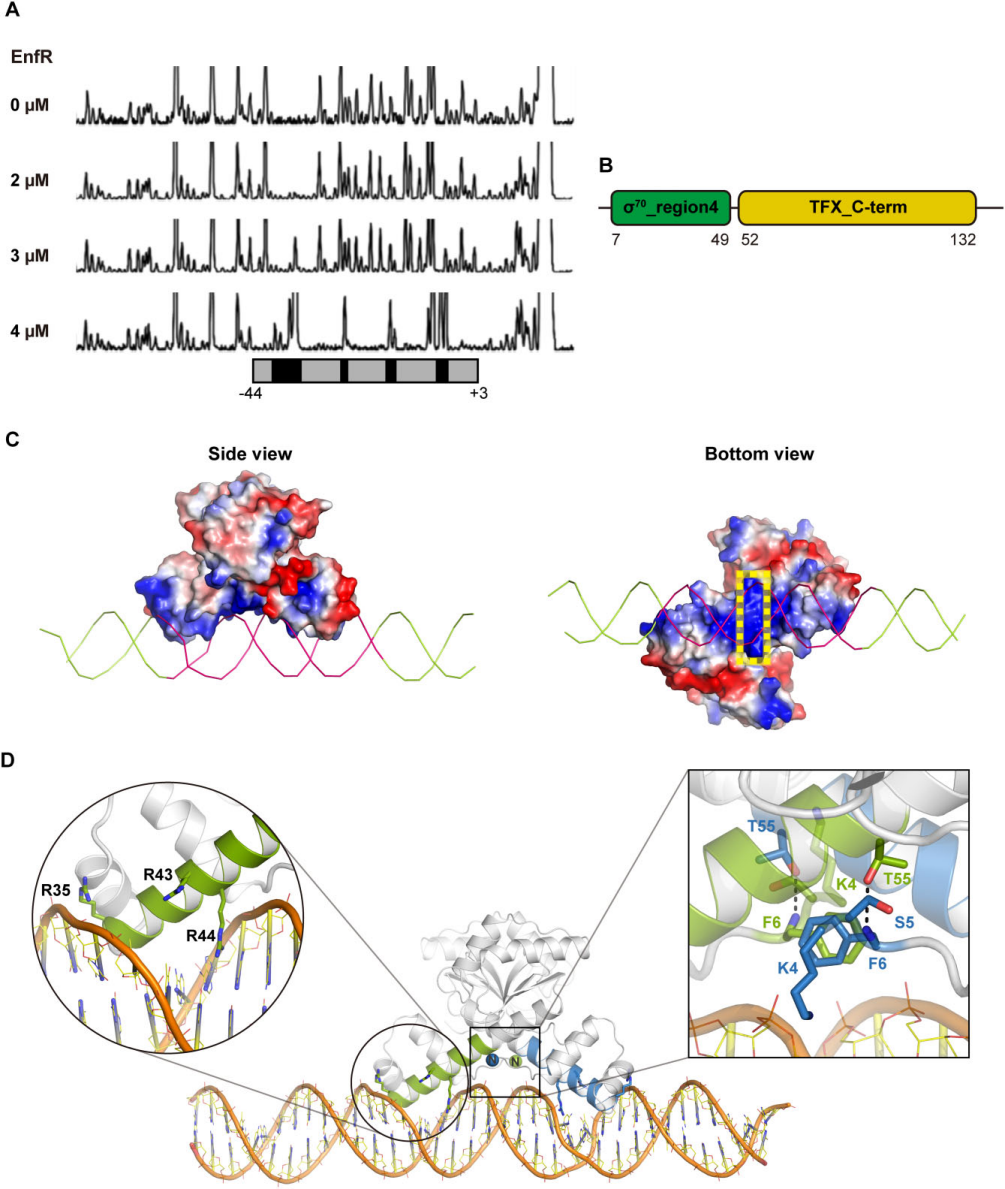
*In* *silico* model of the EnfR/DNA complex. (**A**) DNaseI footprinting analysis of EnfR-binding site in the promoter of the *codh* gene cluster. The protected region by EnfR and the susceptible region to DNaseI are represented by grey and black boxes, respectively. (**B**) Domain architecture of EnfR annotated by *Pfam*. (**C**) Surface representation of the EnfR dimer with the electrostatic potentials in the *in silico* complex model. Positive, negative and neutral electrostatic potentials are colored blue, red and white, respectively. The 36-bp target DNA is represented as a light green ribbon and the minimal EnfR-binding region (5′-AATCTTTTTGTTTACATT-3′) is indicated by pink. The N-terminal loop (residues 1–6, referred to as the *N*-6 region) is indicated by a yellow dotted square. (**D**) The interfaces between the dimeric EnfR and the 36-bp DNA. *Left circle*: DNA-contacting residues (Arg35, Arg43 and Arg44) are shown in green sticks. *Right square*: the residues in the *N*-6 region are also presented by sticks. Hydrogen bonds between the side chain hydroxyl group of Thr55 in a monomer and the backbone -NH group of Phe6 in another monomer are presented by black dotted lines. For clarity, the DNA-binding regions in the dimeric EnfR are colored in green and blue for each monomer.

To elucidate the DNA-recognition mode of EnfR, first, we mapped the binding sequence for EnfR in the *codh* promoter region by using the DNase I protection assay. In the previous study, it was confirmed that the purified EnfR binds specifically to the 150-bp long DNA probe containing the promoter region of *codh* gene cluster (24). When the same DNA probe was incubated with the purified EnfR protein, a region extended from –44 to +3 (centered at –21 from the translation start site of TON_1017, a gene expressed polycistronically with the *codh* gene TON_1018) (13,48) was protected from DNase I digestions (Figure 3A). Inspection of this sequence revealed a 36-bp pseudo-palindromic inverted repeat (5′-cCaTCtTAaAATcTttttgtttAcATTaTAcGAgGt-3′); 18-bp minimal binding region is underlined. Considering the dimeric structure of EnfR, this pseudo-palindromic sequence is highly likely to be a target site for EnfR-binding.

Afterward, we built an *in silico* model structure of the EnfR/DNA complex to reveal the DNA-recognition mode of EnfR in structural aspects. Among structural homologs searched by the *Dali* server (Supplementary Table S7), there were several RNAP σ^70^-like σ factors that are necessary for the initiation of transcription in bacteria. Consistently, according to the Pfam analyses, the α2-turn-α3 HTH motif (residues 7–49) in EnfR corresponds to the region 4 helix-turn-helix (r4-HTH) motif of RNAP σ^70^ (Figure 3B). The α2-turn-α3 HTH motif of EnfR is nicely overlapped onto the r4-HTH motif of *Thermus aquaticus* σ^A^ fragment with the r.m.s. deviation of 0.587 Å for Cα atoms of residues 7–49 (Supplementary Figure S4). Since the r4-HTH motif is responsible for DNA recognition (49), it would appear that the α2-turn-α3 HTH motif is the DNA-binding site in EnfR. In the dimeric structure, two α2-turn-α3 HTH motifs are ∼35.8 Å apart from each other (using the Cα of Glu42 as a reference point). Since the spacing between two successive major grooves is ∼36 Å in the B-form DNA, the two HTH motifs seem to be suitably arranged for the recognition of the target DNA sequence. Based on these perspectives, the complex model structure was generated using the *HADDDOCK* v2.4 program (see the Materials and Methods section).

The resulting complex model is quite reasonable considering electrostatic complementarity and favorable interactions between EnfR and DNA (Figure 3C and D). As shown in Figure 3C, the positively-charged electrostatic potential of the DNA-binding interface in EnfR is adequate to accommodate the negatively-charged sugar-phosphate backbone of DNA. The N-terminal loop (residues 1–6, referred to as the *N*-6 region hereafter) in the two monomers is situated in such a way to make contact with the minor groove (Figure 3C). In addition, the N-terminal part of the stem helix (α3) in the HTH motifs fits into the major groove with three arginine residues (Arg35, Arg43 and Arg44) interacting with DNA. Arg43 and Arg44 extend their side chains toward the bases in the major groove, and Arg35 makes contact with the phosphorous backbone of DNA (Figure 3D). It should be noted that the guanidino group of arginine residues is well known to interact with the base and the backbone of DNA in the protein-DNA complexes (50).

**Figure 4.**
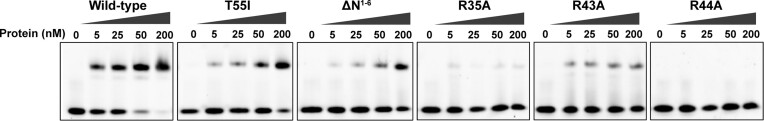
DNA-binding activity of EnfR variants. EnfR (0 ∼ 200 nM) was incubated with the Cy5-labeled 36-bp DNA (5 nM). Δ*N*^1–6^ indicates a mutant with the *N*-6 region deleted.

To verify the *in silico* complex model, we made four mutant proteins (a mutant with the R35A replacement, a mutant with a R43A replacement, a mutant with a R44A replacement, and a mutant with N-terminal six residues deleted (Δ*N*^1–6^)). Notably, the three arginine residues in the HTH motif (Arg35, Arg43, and Arg44) and the N-terminal six residues are directly involved in DNA-binding in the complex model. Point mutations introduced into the solvent-exposed arginine residues and the deletion of the *N*-6 region at the N-terminus cannot affect the dimeric nature of EnfR, which was verified by AUC experiments in which all the mutants were revealed to be dimers (Supplementary Figure S5). The DNA-binding activities of the four mutant proteins (the R35A mutant, the R43A mutant, the R44A mutant, and the Δ*N*^1–6^ mutant) were analyzed by the electrophoretic mobility shift assay (EMSA) with the 36-bp target DNA sequence. In the case of the R35A and R43A mutants, their DNA-binding activities were drastically decreased, and the R44A mutant seemed to lose the DNA-binding activity (Figure 4). These observations, together with the defect of the Δ*N*^1–6^ mutant in DNA-binding (Figure 4), demonstrated that the *N*-6 region and the HTH motif take part in DNA recognition as suggested by the *in silico* complex model.

**Figure 5.**
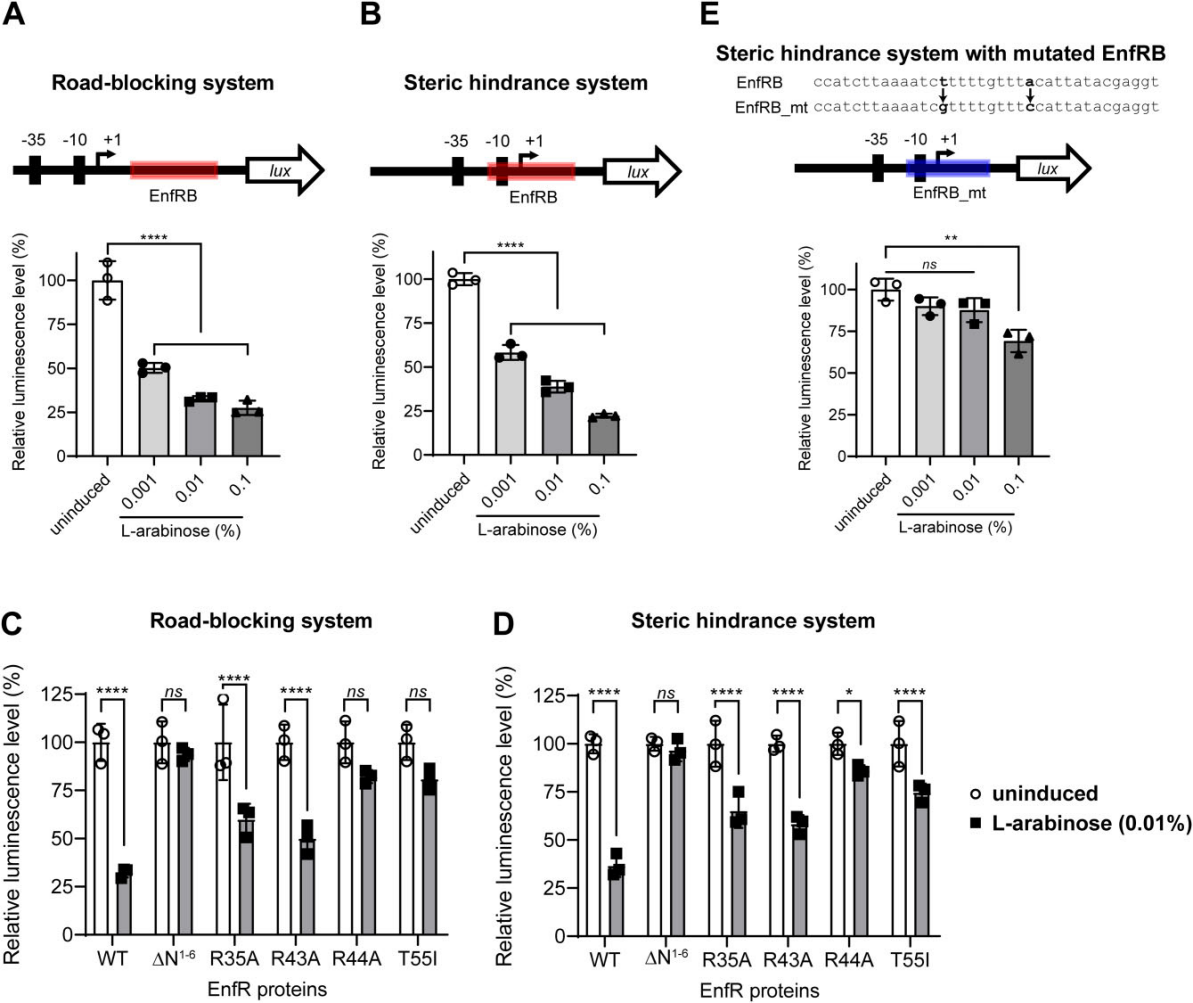
Verification of the EnfR-mediated repression *in vivo*. (A and B) EnfR-mediated repression of the reporter luciferase gene expression was verified either in the road-blocking system (**A**) or the steric hindrance system (**B**). The EnfR-binding site is located at very downstream of or at a partially overlapping position, respectively, with the –10 box for bacterial RNA polymerase. (C and D) Various mutant EnfRs were induced with 0.01% (v/v) of l-arabinose, and their repressor function was examined via luciferase reporter gene assay using road-blocking (**C**) or steric hindrance system (**D**). (**E**) EnfR-mediated repression was significantly dampened if the EnfR-binding sequence in the promoter was mutated (T14G-A23C). WT indicates the wild-type EnfR. For A, B and E, a schematic diagram of each promoter is shown above the graph. The statistical significance was determined by ordinary one-way ANOVA with multiple comparisons (A, B and E) or by multiple t-tests (C and D) (*ns*, not significant; ****P< 0.0001; **P< 0.01; *P< 0.05). EnfRB, EnfR-binding site; EnfRB_mt, Enf-binding site with mutation.

For *in vivo* validation of the *in vitro* mutational studies, we developed two reporter systems: (i) the road-blocking system, of which the target DNA sequence is located between a bacterial constitutive promoter (J23117) (40) and a transcriptional start site of the luciferase (*lux*) operon and (ii) the steric hindrance system, of which the target sequence is partially overlapped with another type of bacterial constitutive promoter (J20110) (Figure 5A and B) (40). The binding of EnfR to the target sequence *in vivo* would interfere with the proceeding or binding of bacterial RNAP in the road-blocking or steric hindrance system, respectively. Indeed, when EnfR was induced via arabinose (Figure 5A and B), the luminescence levels were significantly reduced in both systems in an arabinose concentration-dependent manner. If the wild-type EnfR was induced with 0.01% (v/v) of arabinose in either the road-blocking or steric hindrance system, a relative luminescence level was about 35% compared to the uninduced condition. When R35A, R43A, R44A or Δ*N*^1–6^ was induced, the relative luminescence levels were in the range of 55–85%, which indicated that all the tested mutants have defects in DNA binding as shown in EMSA and that the three arginine residues and the *N*-6 region contribute to DNA-binding (Figure 5C and D).

Furthermore, to examine the effect of base substitutions on EnfR-binding, four base pairs with palindromic relationships in the target DNA sequence were changed; A10-T27 → C10- G27, A11-T26 → C11-G26, T12-A25 → G12-C25 and T14-A23 → G14-C23 (Supplementary Figure S6A and Table S3). Then, the influence of the base substitution on EnfR-binding was examined by EMSA. Among the four substitutions, the T14G-A23C substitution was remarkable in the reduction of EnfR-binding (Supplementary Figure S6B), which was confirmed by the luciferase reporter assay. Compared to the uninduced control, a relative luminescence level in the presence of the T14G-A23C substituted promoter sequence remained at ∼85% and ∼65% after the wild-type EnfR was induced with 0.01% and 0.1% (v/v) of arabinose, respectively (Figure 5E). In contrast, when the original target sequence was used, the relative luminescence level was decreased to about 39% and 22% at 0.01% and 0.1% (v/v) arabinose, respectively (Figure 5B), which indicated that the T14G-A23C substitution reduced the repression activity of EnfR. Collectively, it can be concluded that EnfR loosely binds to the target DNA sequence harboring the T14G-A23C substitution. The road-blocking system was not employed in this base substitution experiment since the T14G-A23C substitution resulted in no expression of *lux* operon, probably due to the formation of a stable secondary structure in the 5′ untranslated region of the transcript.

### Structural explanation for the defect of the T55I mutant in DNA-binding

**Figure 6.**
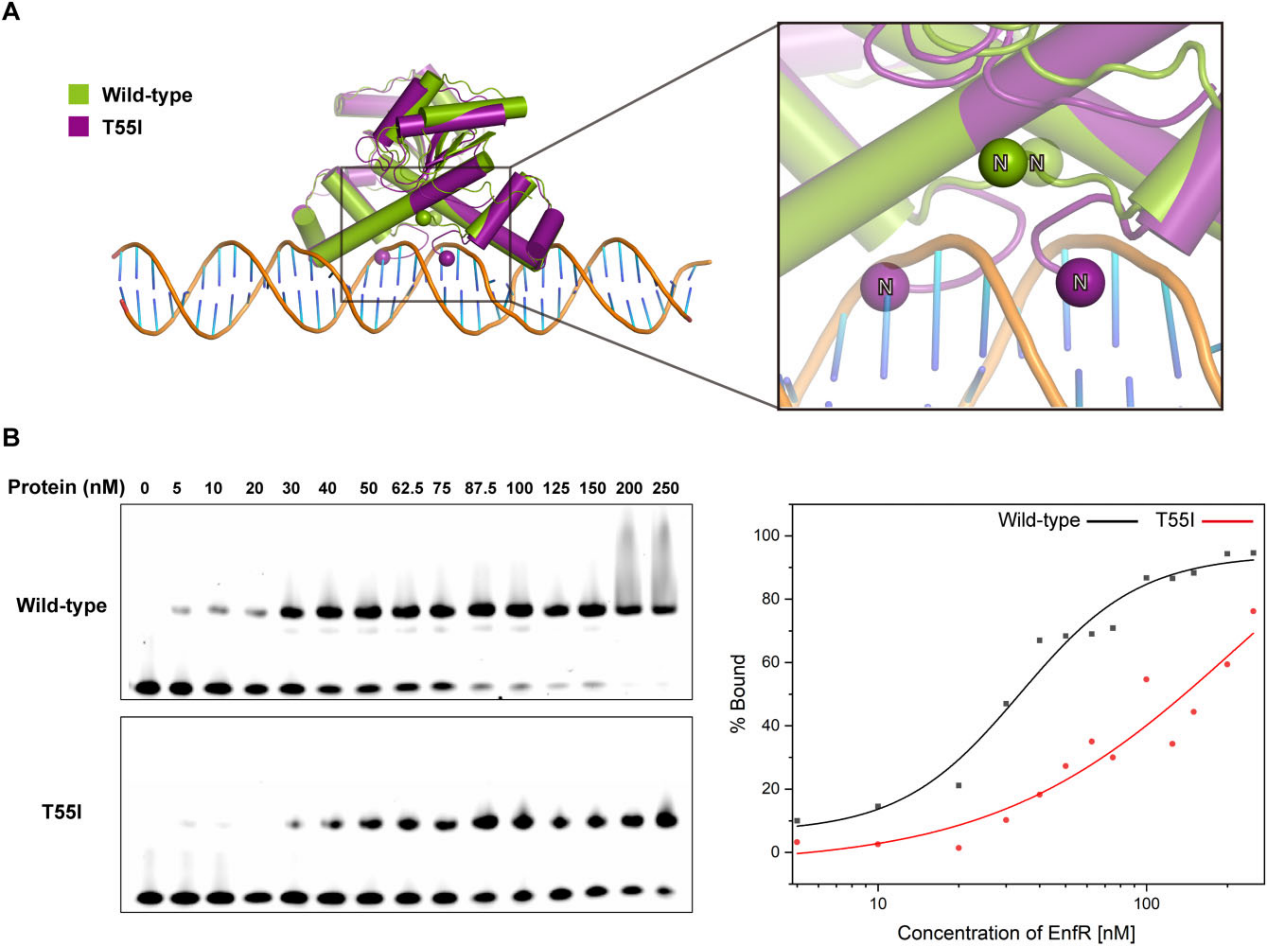
Effect of the T55I mutation on the DNA-binding activity of EnfR. (**A**) The crystal structure of the T55I mutant (purple) was superposed onto the wild-type (green) in the EnfR-DNA complex model. To clearly understand the movement of the *N*-6 region, N-termini are represented by spheres, and all α-helices are shown in cylinders. (**B**) Electrophoretic mobility shift assay (EMSA) for the wild-type and the T55I mutant. EnfR (0–250 nM) was incubated with the Cy5-labeled 36-bp DNA (5 nM). The fractions of bound 36-bp dsDNA were plotted against EnfR concentrations (0, 5, 10, 20, 30, 40, 50, 62.5, 75, 100, 125, 150, 200 and 250 nM) with a Hill 1 equation. The data from the wild-type and the T55I mutant are colored black and red, respectively.

**Figure 7.**
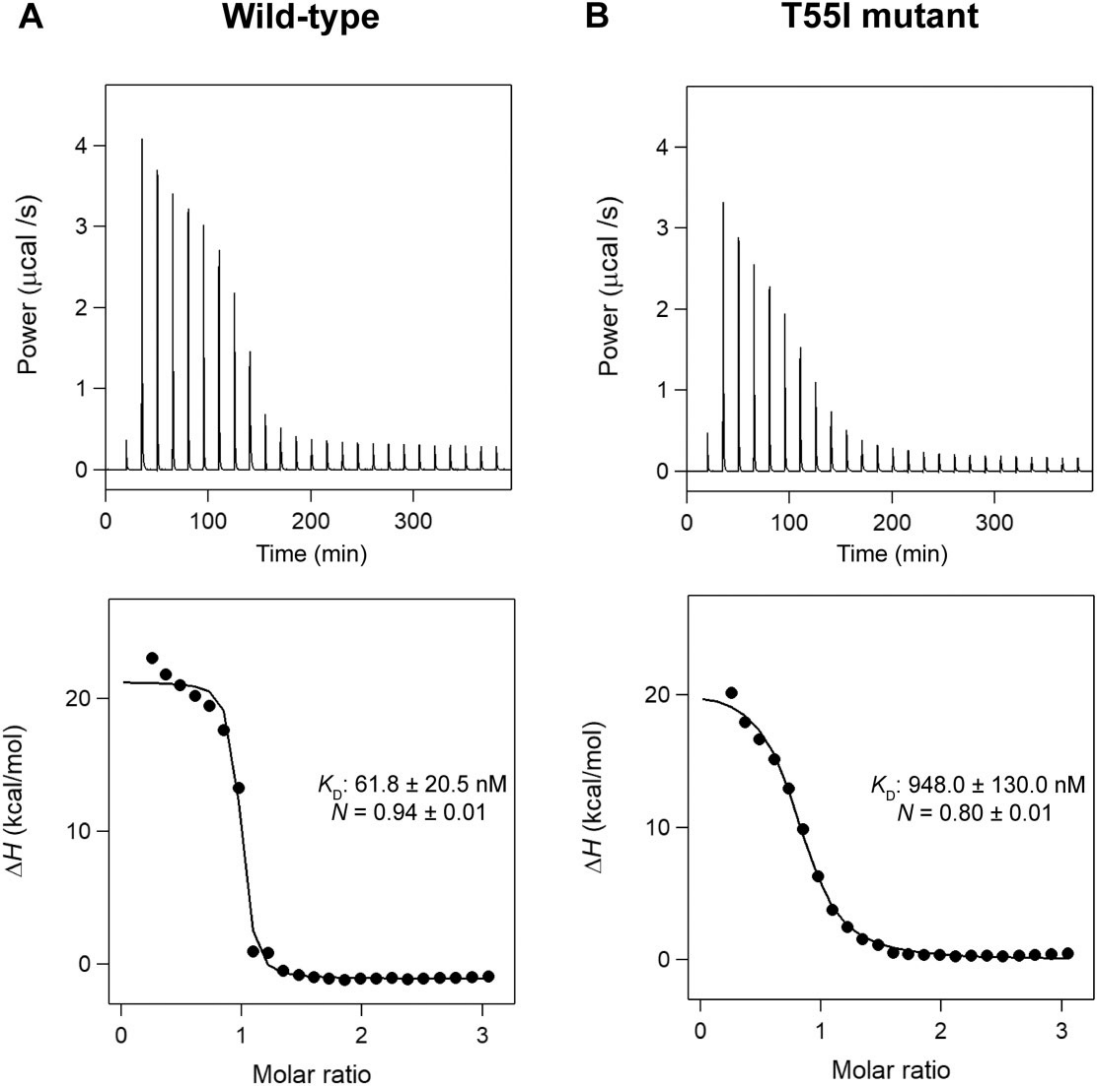
Calorimetry-based characterization of intermolecular interactions between EnfR and 36-bp. (A and B) ITC thermograms (upper) and binding isotherms (lower) observed upon titration of 36-bp into wild-type (**A**) and mutant EnfR (**B**). Closed circles and solid lines represent the normalized values of the enthalpy change (Δ*H*) and fit curves, respectively.

The transcription level of genes in the *codh* cluster was reported to be affected by the T55I mutation in EnfR (24). To examine the influence of the T55I mutation on the DNA-binding activity of EnfR, EMSA was performed. The Cy5-labelled target DNA sequence was titrated as the concentration of the wild-type and T55I mutant increased. In both cases, single retarded bands were observed, and their intensities were increased in a concentration-dependent manner (Figure 6B), which indicates only one dimeric EnfR binds to the target sequence. Remarkably, the T55I mutation seemed to cause a defect in the DNA-binding activity. The intensity of the retarded band in the presence of the T55I mutant was lower than that of the wild-type in EMSA (Figure 6). Consistently, when the T55I mutant was induced in the luciferase reporter assay, the luminescence level was increased compared to in the presence of the wild-type (Figure 5C and D). For quantitative measurements of the negative effect of the T55I mutation on DNA-binding activity, we determined the dissociation constant (*K*_D_) of the wild-type and the T55I mutant towards the target DNA sequence by isothermal titration calorimetry (ITC). The *K*_D_ values of the wild-type and the T55I mutant were calculated to be 61.8 ± 20.5 and 948.0 ± 130.0 nM, respectively, clearly indicating that the DNA-binding affinity of the mutant is much lower (over fifteen-fold) than that of the wild-type. The stoichiometry parameters (*N*) were 0.94 ± 0.01 for the wild-type and 0.80 ± 0.01 for the T55I mutant, which confirmed that one dimeric EnfR binds to the 36-bp target DNA sequence (Figure 7).

**Figure 8.**
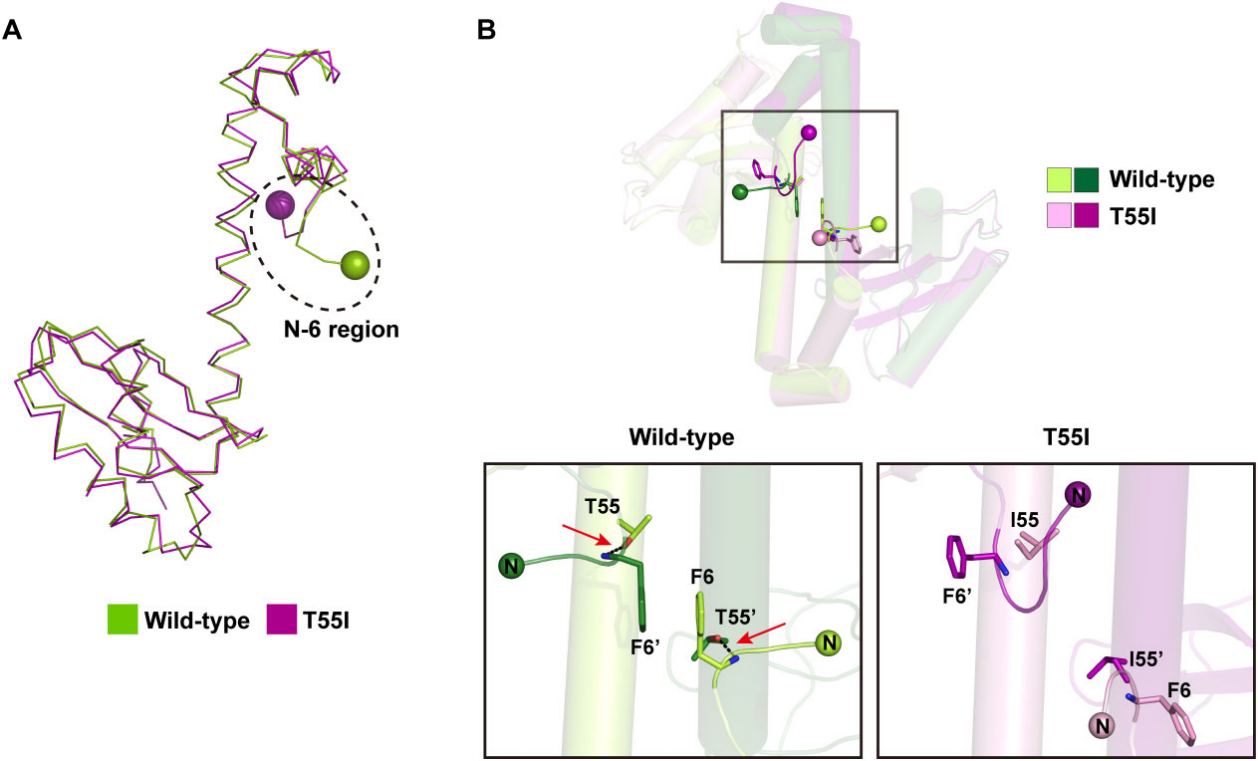
Conformational changes induced by the T55I mutation. (**A**) Superposition of monomeric structures of the wild-type (green) and the T55I mutant (purple). A black dotted circle indicates the conformational changes in the *N*-6 region between the wild-type and the T55I mutant. (**B**) *top*: superposition of dimeric structures of the wild-type (each monomer in green colors of different depth) and the T55I mutant (each monomer in purple colors of different depth). *bottom*: black dotted lines pointed out by red arrows indicate hydrogen bonds between the side chain hydroxyl group of Thr55 in a monomer and the backbone -NH group of Phe6 in another monomer. N-termini are presented by spheres.

To get structural insights into the reduced DNA-binding activity of the T55I mutant, we determined its 3.4 Å resolution crystal structure (Supplementary Table S1). The overall structures of the monomeric and dimeric T55I mutant are virtually identical to those of the wide-type. The r.m.s. deviations of monomers and dimers between the mutant and the wild-type are 0.827 and 0.941 Å, respectively, for all Cα atoms (Figure 8). Despite this structural resemblance, however, the T55I mutant has a remarkable structural difference in the orientation and conformation of the *N*-6 region that is engaged in DNA recognition. In the wild-type dimer, the side chain hydroxyl group of Thr55 in a monomer is hydrogen-bonded to the backbone –NH group of Phe6 in another monomer, which directs the *N*-6 region to extend out of the main body (Figure 8). Two benzyl side chains of Phe6 in the *N*-6 regions of two monomers form π–π stacking interaction to stabilize the observed conformation of the *N*-6 region. In contrast, in the case of the T55I mutant, the *N*-6 region runs in a different direction due to the loss of the hydrogen bond, which is the consequence of the isoleucine replacement at position 55 (Figure 8). In this conformation of the two *N*-6 regions, the two phenylalanine residues cannot form π–π stacking interactions.

To elucidate structural determinants for the dramatic effect of the T55I mutation on DNA-binding activity, we made a complex model between the T55I mutant and DNA by substituting the wild-type structure with the T55I mutant structure in the *in silico* EnfR/DNA complex model. In the resulting complex model, it was obvious that the *N*-6 region of the mutant clashes with DNA (Figure 6A). Consequently, it can be concluded that the altered conformation of the *N*-6 region induced by the T55I mutation disturbs the DNA-binding activity of the mutant, and the conformation of T55I mutant is not adequate in DNA-binding.

### EnfR is a repressor regulating the expression of the *codh* gene cluster

The archaeal core promoter region contains two consensus sequences, A–T rich TATA-box and purine-rich BRE (25). Transcription factor B (TFB) and TATA-binding protein (TBP) bind to BRE and TATA-box, respectively, and recruit RNAP to form pre-initiation complexes (PIC) and start transcription (51). When we analyzed the 36-bp target DNA sequence resulting from the DNase I protection assay, there was a TATA box-like sequence consisting of TTATA in 15–19 bp upstream from the translation start site of the *codh* gene cluster. In other words, EnfR binds to the TATA box-like region. Notably, transcription repressors in archaea usually bind to sequences downstream of BRE including TATA-box regions to block the formation of PIC (52). Therefore, the sequence analysis for the promoter region of the *codh* gene cluster, together with the reduced affinity of the T55I mutant toward the target DNA sequence, suggests that EnfR acts as a transcriptional repressor that controls the expression level of the *codh* gene cluster.

### Changes in diverse gene expression in the MC11 strain promote the cell growth and H_2_ production under carboxydotrophic condition

**Figure 9.**
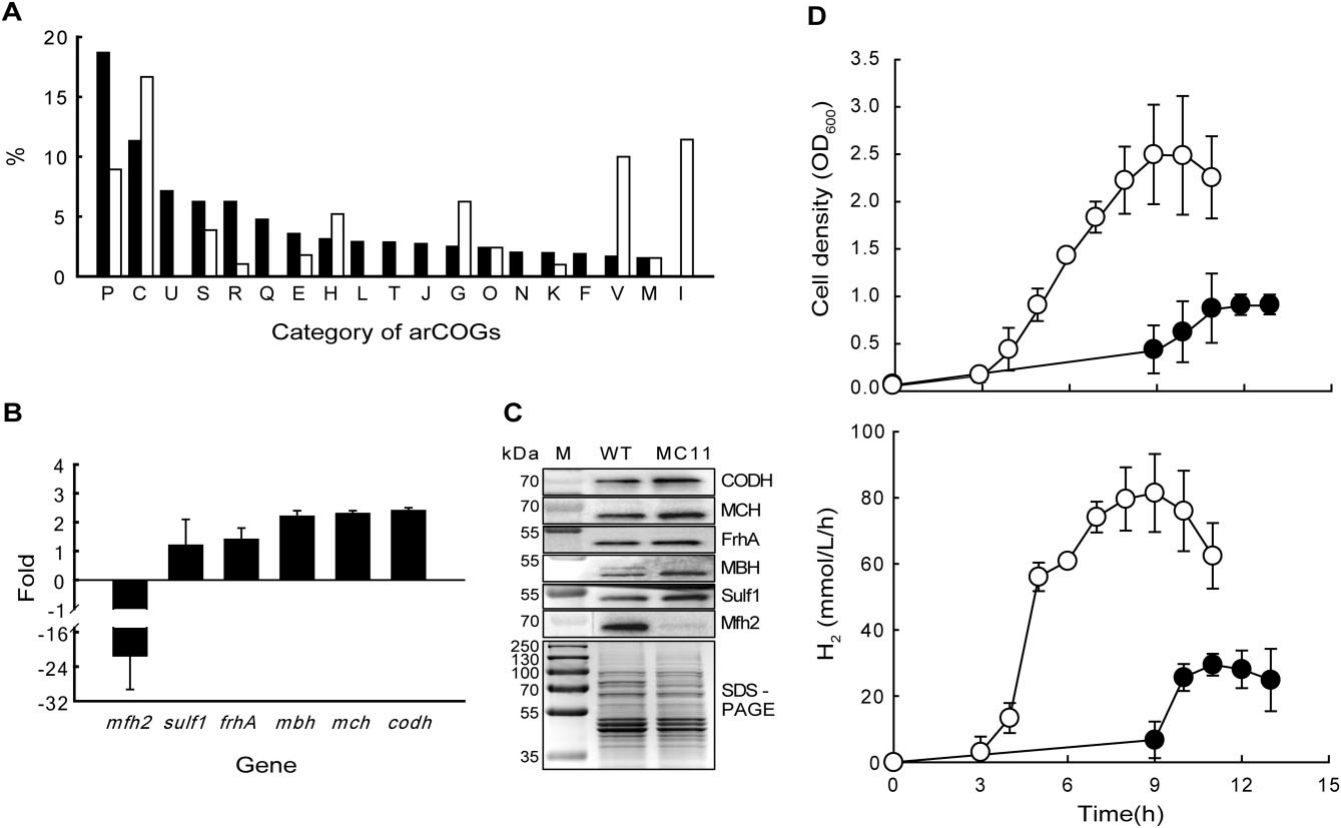
Transcriptome and phenotypic analysis of the MC11 strain. (**A**) Distribution of differentially expressed genes (DEGs) in the archaeal clusters of orthologous genes (arCOGs). The number indicates the proportion of genes that show significant (≥2-fold) increase (filled bars) or decrease (empty bars) in each group. The one letter code for arCOG categories is as follows: P, Inorganic ion transport and metabolism; C, Energy production and conversion; U, Intracellular trafficking, secretion and vesicular transport; S, Function unknown; R, General function prediction only; Q, Secondary metabolite biosynthesis, transport and catabolism; E, Amino acid transport and metabolism; H, Coenzyme transport and metabolism; L, Replication, recombination and repair; T, Signal transduction mechanisms; J, Translation, ribosomal structure and biogenesis; G, Carbohydrate transport and metabolism; O, Posttranslational modification, protein turnover and chaperones; N, Cell motility; K, Transcription; F, Nucleotide transport and metabolism; V, Defense mechanisms; M, cell wall/membrane/envelope biogenesis and I, Lipid transport and metabolism. (B, C) Expression profile of (de)hydrogenases in transcriptome analysis (**B**) and western blotting analysis (**C**). The fold change represents the ratio of the expression level of each gene identified from the triplicate analysis between the wild-type and the MC11 strain. (**D**) Cell growth and H_2_ production of the wild-type (closed circles) and the MC11 strain (open circles) strains with 100% CO. The data for the wild-type strain was adapted from our previous report (35). Error bars indicate the standard deviations of three independent cultivations of the MC11 strain in this study.

According to our previous reports (24,35), the T55I mutation in EnfR (156T strain) and the deletion of EnfR (Δ1525 strain) led to significant increase of CO oxidation activity and H_2_ production rate, which was accompanied by the increased expression of the *codh* gene cluster. In addition, we have also shown that Δ1525 led to expression changes in various genes (35). To further explore the inherent role of EnfR, we performed comparative transcriptome analyses between the wild-type and the MC11 strain harboring the T55I mutation. Sequencing reads were mapped to 1914 protein-coding genes. Among them, significantly (≥2-fold) up- or down-regulated genes in the MC11 strain were 101 (5.3%) and 77 (4.0%) genes, respectively (Supplementary Table S9 and S10). The expression of the gene encoding EnfR (TON_1525) also increased 2.9-fold, suggesting the potential for self-regulation. The differentially expressed genes (DEGs) were assigned to multiple groups of the archaeal clusters of orthologous genes (arCOGs) (Figure 9A).

Groups P (inorganic ion transport and metabolism) and C (energy production and conversion) had high proportions for up-regulated DEGs (18.7 and 11.3%). On the other hand, groups C (energy production and conversion), I (lipid transport and metabolism) and V (defense mechanisms) showed high proportions for down-regulated DEGs (16.7, 11.8 and 10%). It is noteworthy that group C showed an amphoteric pattern with a high proportion of both up- and down-regulated DEGs. Since hydrogenases are known to play an important role in energy metabolism by generating electrochemical ion gradient along with H_2_ production or in H_2_ metabolism by disposing of reducing equivalents in hyperthermophilic archaea such as *Pyrococcus* and *Thermococcus* (34,53,54), the expression change of the genes encoding them was further analyzed. Transcriptome analyses showed that many hydrogenase genes and related dehydrogenase genes were up-regulated in the MC11 strain: *frhA* encoding Frh hydrogenase α subunit (1.4-fold), *mbh* encoding membrane-bound hydrogenase (2.2-fold), *mch* encoding membrane-bound carbon monoxide-dependent hydrogenase (2.3-fold), *sulf1* encoding soluble hydrogenase Sulf1 (1.2-fold) and *codh* encoding carbon monoxide dehydrogenase (2.4-fold) (Figure 9B). On the contrary, the expression level of *sulf2* gene encoding soluble hydrogenase Sulf2 was 0.7-fold down-regulated (data not shown), and *mfh2* encoding membrane-bound formate-dependent hydrogenase was significantly down-regulated by 21.5-fold in the MC11 strain (Figure 9B). Expression changes at the transcription level were consistent with the data at the protein level obtained by Western blotting (Figure 9C).

Previously, resting cell assay revealed enhanced CO oxidation activity of the MC11 strain (24). Considering the up-regulation of *codh* and *mch* gene expression, it was assumed that carboxydotrophic growth of the MC11 strain would be improved. According to bioreactor experiments, significant enhancement of cell growth and H_2_ production of the MC11 strain was observed (Figure 9D). The maximum cell density and H_2_ production rate were increased 2.5- and 2.8-fold, respectively, compared to those of the wild-type strain.

In this study, whole transcriptome analysis revealed the potential of EnfR to control expression of a broad array of genes at the transcription level. Sulfur-responsive regulator, SurR and *Thermococcales* glycolytic regulator, Tgr/TrmBL1 (55,56), are well-known global regulators in *Thermococcales* species to regulate diverse gene expression associated with energy conservation and central metabolic pathway for glycolysis and gluconeogenesis, respectively (56,57). It is not yet clear whether EnfR can have pleiotropic effects on various cellular processes. However, the distribution of large numbers of DEGs for a wide range of arCOGs implicates that EnfR might have a role in regulating the expression of diverse genes and has the potential to influence various metabolic processes. To investigate whether EnfR binds to promoter regions of DEGs, we selected two up-regulated genes TON_1582 and TON_0537 that encode a Na^+^/H^+^ antiporter and sulfhydrogenase β subunit SulfI, respectively. In addition, one down-regulated gene TON_1563 encoding formate dehydrogenase was selected. According to EMSA experiments (Supplementary Figure S7), retarded bands for the promoter DNA sequences of the three genes were observed in the presence of EnfR, and they disappeared when cold DNA was added, which showed the specific association of EnfR with the three promoter regions. To verify the possibility of EnfR to act as a global regulator, further studies including chromatin immunoprecipitation sequencing and investigation of phenotypic changes of the EnfR mutants under various culture conditions are needed.

## Supplementary Material

gkad699_Supplemental_Files

## Data Availability

The atomic coordinates and structure factors of the final models have been deposited in the Protein Data Bank with ID codes 8HNO (the wild-type), 8HNP (the T55I mutant). RNA sequence data were submitted to the NCBI Gene Expression Omnibus (GEO) database (http://www.ncbi.nlm.nih.gov/geo) with accession code GSE200806.
