# Supplementary material for: An archaeal transcription factor EnfR with a novel ‘eighth note’ fold controls hydrogen production of a hyperthermophilic archaeon Thermococcus onnurineus NA1
Source: Nucleic Acids Res. 2023 Aug 31;51(18):10026–40. doi: 10.1093/nar/gkad699 (PMC10570040; doi:10.1093/nar/gkad699)
Supplement: gkad699_Supplemental_Files [file gkad699_supplemental_files.zip › Second_Legends_of_Supplemenatry_Data.docx]

**Supplementary data**

**An archaeal transcription factor EnfR with a novel ‘eighth note’ fold controls hydrogen production of a hyperthermophilic archaeon *Thermococcus onnurineus* NA1**

Da-Woon Bae^1,#^, Seong Hyuk Lee^2,#^, Ji Hye Park^4^, Se-Young Son^1^, Yuxi Lin^6^, Jung Hyen Lee^4^, Bo-Ram Jang^5^, Kyu-Ho Lee^5^, Young-Ho Lee^6-10^, Hyun Sook Lee^2,3^, Sung Gyun Kang^2,3^, Byoung Sik Kim^4^, Sun-Shin Cha^1,*^

^1^Department of Chemistry & Nanoscience, Ewha Womans University, Seoul, 03760, Republic of Korea

^2^Marine Biotechnology Research Center, Korea Institute of Ocean Science and Technology, Busan, South Korea

^3^Department of Marine Biotechnology, KIOST School, University of Science and Technology, Daejeon, South Korea

^4^Department of Food Science and Biotechnology, Ewha Womans University, Seoul, 03760, Republic of Korea

^5^Department of Life Science, Sogang University, 35 Baekbeom-Ro, Mapo-Gu, Seoul, South Korea.

^6^Research Center for Bioconvergence Analysis, Korea Basic Science Institute (KBSI), 8 Chungbuk 28119, Republic of Korea

^7^Bio-Analytical Science, University of Science and Technology, Daejeon 34113, Republic of Korea

^8^Graduate School of Analytical Science and Technology, Chungnam National University, Daejeon 34134, Republic of Korea

^9^Department of Systems Biotechnology, Chung-Ang University, Gyeonggi 17546, Republic of Korea

^10^Frontier Research Institute for Interdisciplinary Sciences, Tohoku University, Miyagi 980-8578, Japan

# These authors equally contributed to this work

*Address correspondence to Sun-Shin Cha, [chajung@ewha.ac.kr](mailto:chajung@ewha.ac.kr)

Keywords: *Thermococcus onnurineus* NA1, Archaeal transcription factor, Crystal structure, Hydrogen production, Tfx DNA-binding protein family

**Supplementary Figure S1.** **Topology diagram for the note-head domain (NHD).** NHD is highlighted with yellow-dashed circle in the left panel. In the right panel, α-helices and β-strands are presented by purple cylinders and lightgrey arrow, respectively. The alphabet subscripts indicate the order of secondary structural elements represented by Greek letters.

**Supplementary Figure S2.** **SV-AUC of an EnfR mutant harboring three point mutations (A52W, I58G, and I62G).** *Left* panel: raw sediment velocity profiles recorded using absorbance at 280 nm. *Right* panel: the distribution of sedimentation coefficient *c*(s) from the model. Black arrow indicates the monomeric EnfR.

**Supplementary Figure S3.** **Structural comparison between EnfR and the bacterial RNA polymerase sigma subunit σ_4_^L^.** EnfR and σ_4_^L^ (PDB entry 3HUG chain A) are superposed and colored in lime-green and magenta, respectively. N-and C-termini of EnfR and σ_4_^L^ are presented by blue and red spheres, respectively.

**Supplementary Figure S4.** **Structural similarity between the N-terminal domain of EnfR and** **the bacterial RNA polymerase sigma subunit.** A monomeric EnfR is superposed onto the r4-HTH motif of *Thermus aquaticus* σ^A^ fragment (σ_4_) (PDB entry: 1KU7). The N-terminal HTH motif and the Tfx C-terminal motif of EnfR analyzed by *Pfam* are colored in green and yellow, respectively.

**Supplementary Figure S5.** **Dimeric state of EnfR mutants.** SV-AUC of EnfR mutants. Left panel: raw sediment velocity profiles recorded using absorbance at 280 nm. Right panel: the distribution of sedimentation coefficient *c*(s) from the model. The analytical ultracentrifugation profiles of all EnfR mutants showed they exist as a dimer in solution.

**Supplementary Figure S6.** **The effect of base substitutions on the DNA-binding of EnfR.** (A) EnfR-binding 36-bp target DNA sequence. Capital letters in bold represent pseudo-palindromic inverted repeat DNA sequences, and letters in blue correspond to the minimal EnfR-binding region. (B) EMSA for mutated EnfR-binding sites. EnfR (0 ~ 200 nM) was incubated with Cy5-labeled mutated sequences (5 nM).

**Supplementary Figure S7.** **The binding of EnfR to promoters of DEGs.** 10 nM EnfR was incubated with the Cy5-labeled promoter fragments (10 nM). P_1582_ and P_0537_ indicate promoter regions of TON_1582 encoding Na+/H+ antiporters and TON_0537 encoding sulfhydrogenase beta subunit SulfI, respectively. P_1563_ represents the promoter region of TON_1565, which encodes formate dehydrogenase. Cold means a specific competitor probe, unlabeled 36-bp target DNA of EnfR.

**Supplementary Table S1. X-ray diffraction data and refinement statistics**

**Supplementary Table S2. Primer sequences to amplify the promoter region of the *codh* gene cluster**

Supplementary Table S3. Original and substituted sequences of the EnfR-binding site

**Supplementary Table S4. Primer sequences to amplify the promoter regions of three DEGs**

**Supplementary Table S5.** **Strains and plasmids used in this study**

**Supplementary Table S6.** **Sequences of the DNA fragments containing the engineered constitutive promoter with EnfR-binding site**

**Supplementary Table S7.** **Structural homologs of monomeric EnfR**

**Supplementary Table S8.** **Structural homologs of the note-head domain of EnfR**

**Supplementary Table S9.** **List of 101 genes transcriptionally up-regulated (≥2-fold) in the MC11 strain as compared to the wild-type strain.**

**Supplementary Table S10. List of 77 genes transcriptionally down-regulated (≥2-fold) in the MC11 strain as compared to the wild-type strain.**
