# Supplementary material for: An archaeal transcription factor EnfR with a novel ‘eighth note’ fold controls hydrogen production of a hyperthermophilic archaeon Thermococcus onnurineus NA1
Source: Nucleic Acids Res. 2023 Aug 31;51(18):10026–40. doi: 10.1093/nar/gkad699 (PMC10570040; doi:10.1093/nar/gkad699)
Supplement: gkad699_Supplemental_Files [file gkad699_supplemental_files.zip › Supplementary_Data_FigureS1-S7_TableS1-S8.pdf]

Supplementary data

An archaeal transcription factor EnfR with a novel 'eighth note' fold controls hydrogen production of a hyperthermophilic archaeon *Thermococcus onnurineus* NA1

Da-Woon Bae[1,#], Seong Hyuk Lee[2,#], Ji Hye Park[4], Se-Young Son[1], Yuxi Lin[6], Jung Hyen Lee[4], Bo-Ram Jang[5], Kyu-Ho Lee[5], Young-Ho Lee[6-10], Hyun Sook Lee[2,3], Sung Gyun Kang[2,3], Byoung Sik Kim[4], Sun-Shin Cha[1,*]

[1]Department of Chemistry & Nanoscience, Ewha Womans University, Seoul, 03760, Republic of Korea

[2]Marine Biotechnology Research Center, Korea Institute of Ocean Science and Technology, Busan, South Korea

[3]Department of Marine Biotechnology, KIOST School, University of Science and Technology, Daejeon, South Korea

[4]Department of Food Science and Biotechnology, Ewha Womans University, Seoul, 03760, Republic of Korea

[5]Department of Life Science, Sogang University, 35 Baekbeom-Ro, Mapo-Gu, Seoul, South Korea.

[6]Research Center for Bioconvergence Analysis, Korea Basic Science Institute (KBSI), 8 Chungbuk 28119, Republic of Korea

[7]Bio-Analytical Science, University of Science and Technology, Daejeon 34113, Republic of Korea

[8]Graduate School of Analytical Science and Technology, Chungnam National University, Daejeon 34134, Republic of Korea

[9]Department of Systems Biotechnology, Chung-Ang University, Gyeonggi 17546, Republic of Korea

[10]Frontier Research Institute for Interdisciplinary Sciences, Tohoku University, Miyagi 980-8578, Japan

# These authors equally contributed to this work

*Address correspondence to Sun-Shin Cha, chajung@ewha.ac.kr

Keywords: *Thermococcus onnurineus* NA1, Archaeal transcription factor, Crystal structure, Hydrogen production, Tfx DNA-binding protein family

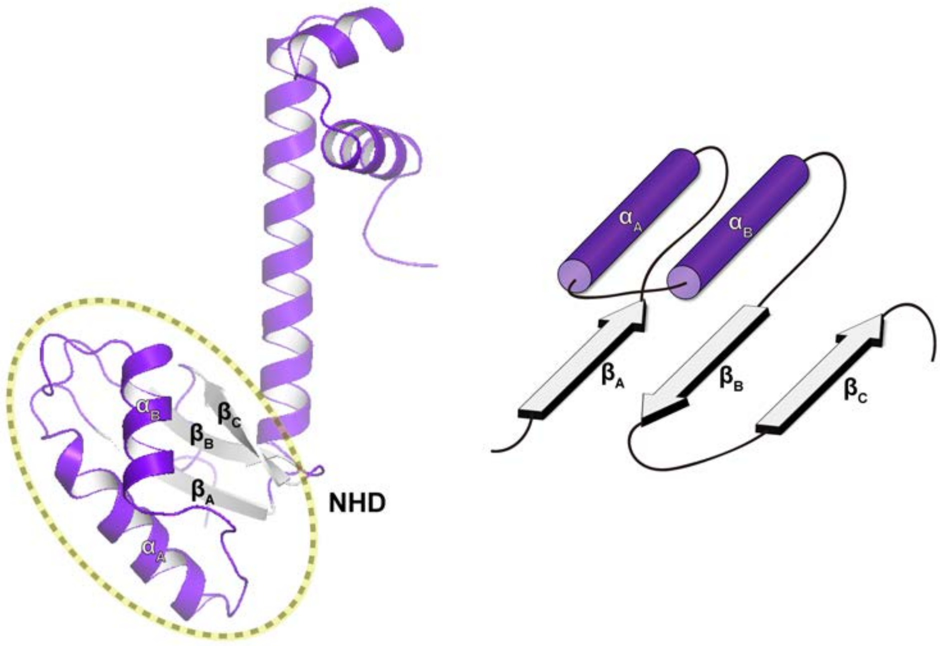

Figure S1. Topology diagram for the note-head domain (NHD). NHD is highlighted with yellow-dashed circle in the left panel. In the right panel, α-helices and β-strands are presented by purple cylinders and lightgrey arrow, respectively. The alphabet subscripts indicate the order of secondary structural elements represented by Greek letters.

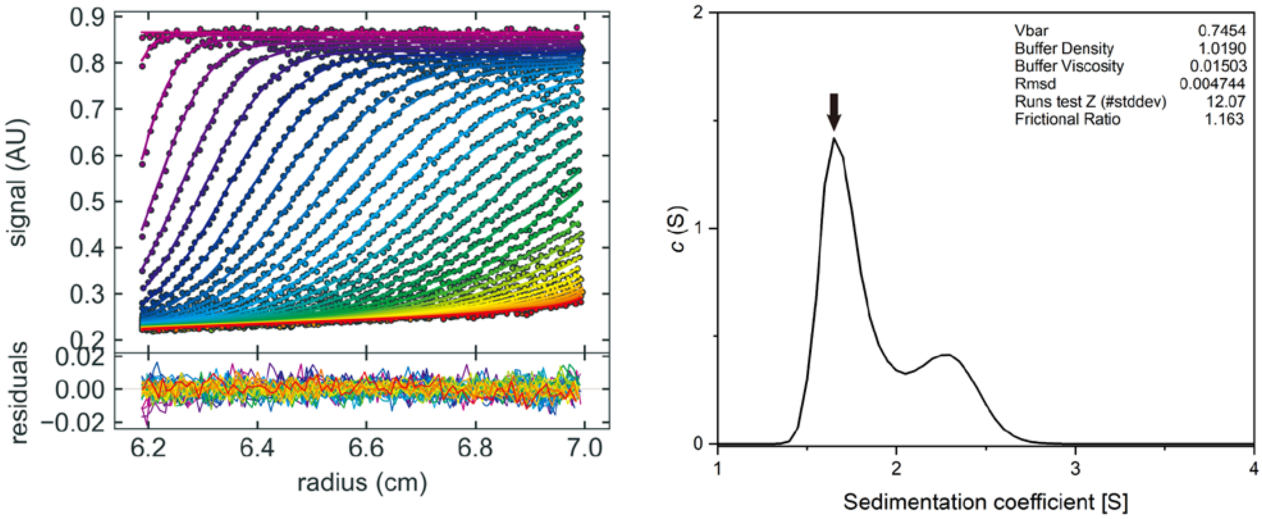

Figure S2. SV-AUC of an EnfR mutant harboring three point mutations (A52W, I58G, and I62G). *Left* panel: raw sediment velocity profiles recorded using absorbance at 280 nm. *Right* panel: the distribution of sedimentation coefficient *c*(s) from the model. Black arrow indicates the monomeric EnfR.

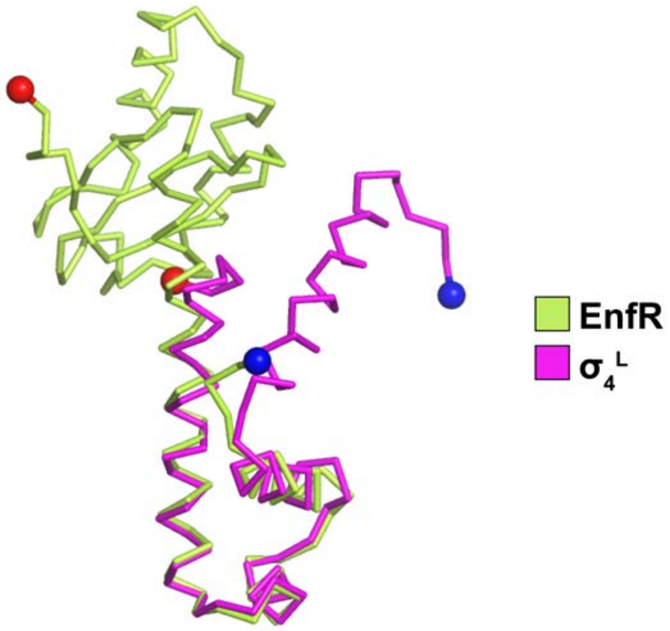

Figure S3. Structural comparison between EnfR and the bacterial RNA polymerase sigma subunit σ_4^L. EnfR and σ_4^L (PDB entry 3HUG chain A) are superposed and colored in lime-green and magenta, respectively. N-and C-termini of EnfR and σ_4^L are presented by blue and red spheres, respectively.

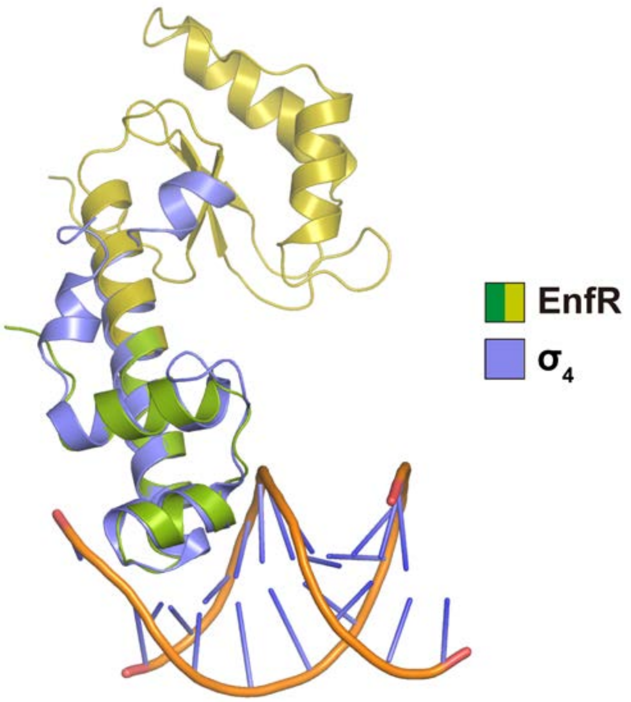

Figure S4. Structural similarity between the N-terminal domain of EnfR and the bacterial RNA polymerase sigma subunit. A monomeric EnfR is superposed onto the r4-HTH motif of *Thermus aquaticus* σ^A fragment (σ_4) (PDB entry: 1KU7). The N-terminal HTH motif and the Tfx C-terminal motif of EnfR analyzed by *Pfam* are colored in green and yellow, respectively.

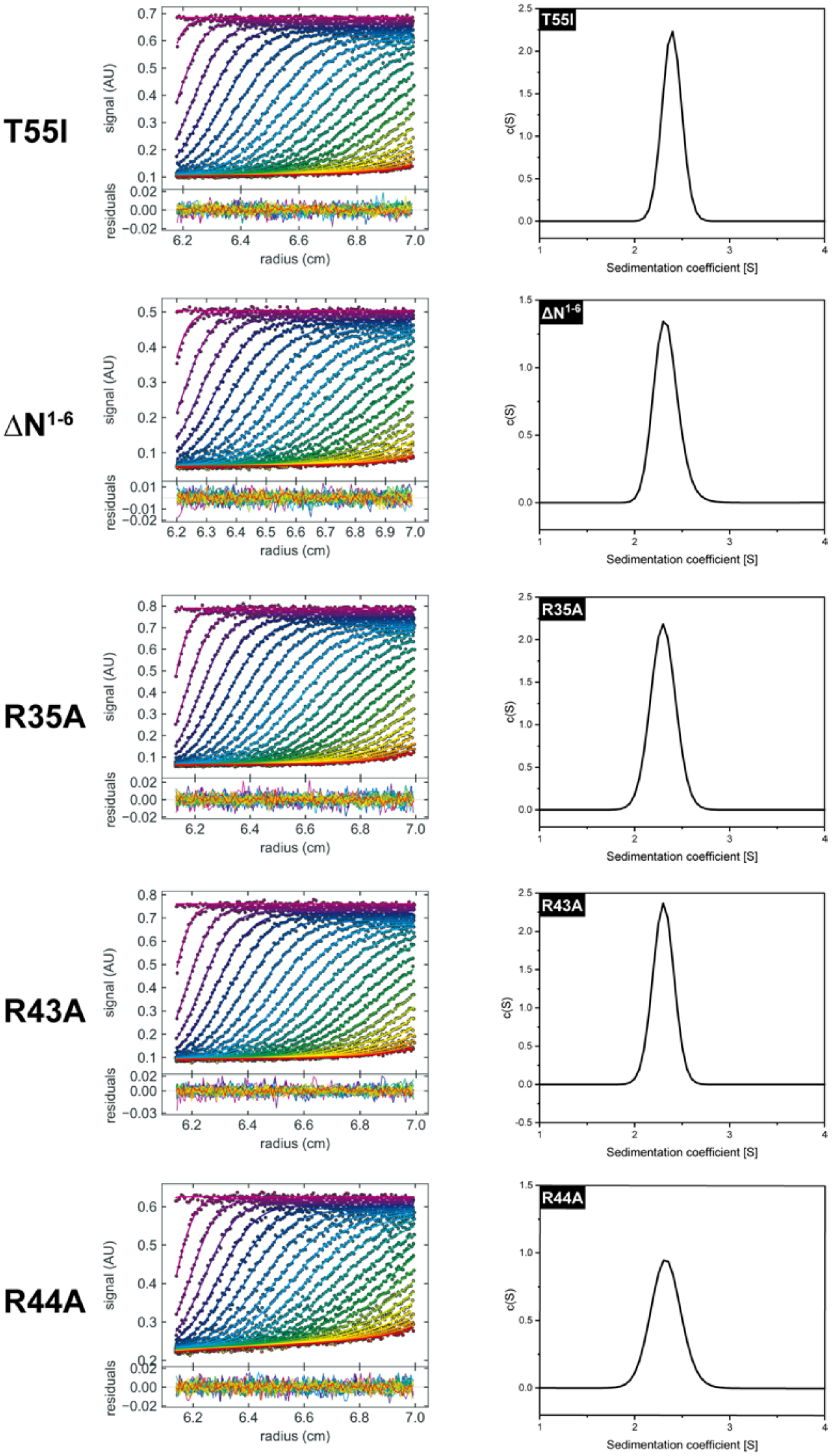
T55I
ΔN1-6
R35A
R43A
R44A
signal (AU)
residuals
radius (cm)
c(S)
Sedimentation coefficient [S]
T55I
ΔN1-6
R35A
R43A
R44A

Figure S5. Dimeric state of EnfR mutants. SV-AUC of EnfR mutants. Left panel: raw sediment velocity profiles recorded using absorbance at 280 nm. Right panel: the distribution of sedimentation coefficient *c*(s) from the model. The analytical ultracentrifugation profiles of all EnfR mutants showed they exist as a dimer in solution.

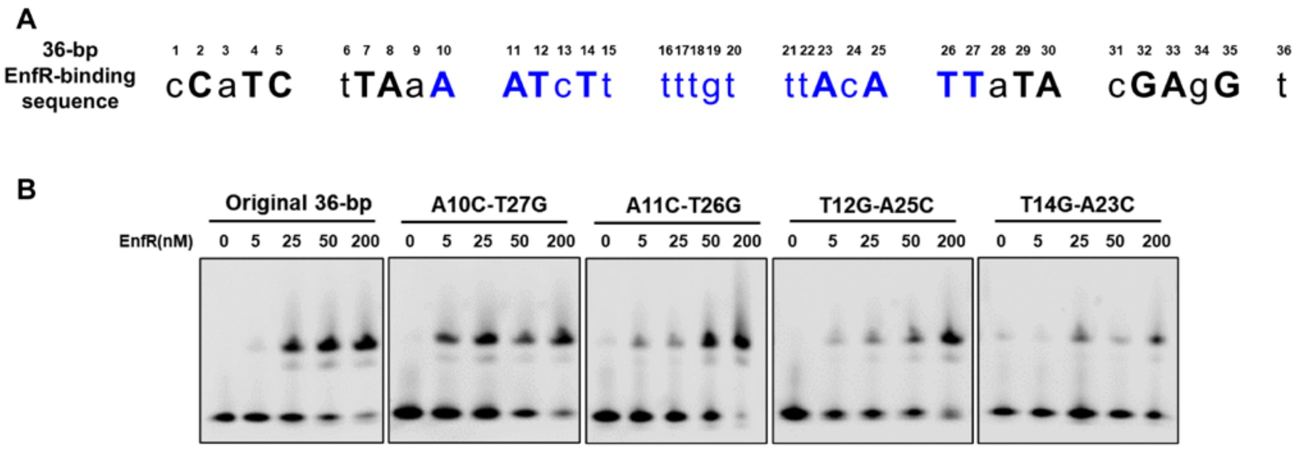

Figure S6. The effect of base substitutions on the DNA-binding of EnfR. (A) EnfR-binding 36-bp target DNA sequence. Capital letters in bold represent pseudo-palindromic inverted repeat DNA sequences, and letters in blue correspond to the minimal EnfR-binding region. (B) EMSA for mutated EnfR-binding sites. EnfR (0 ~ 200 nM) was incubated with Cy5-labeled mutated sequences (5 nM).

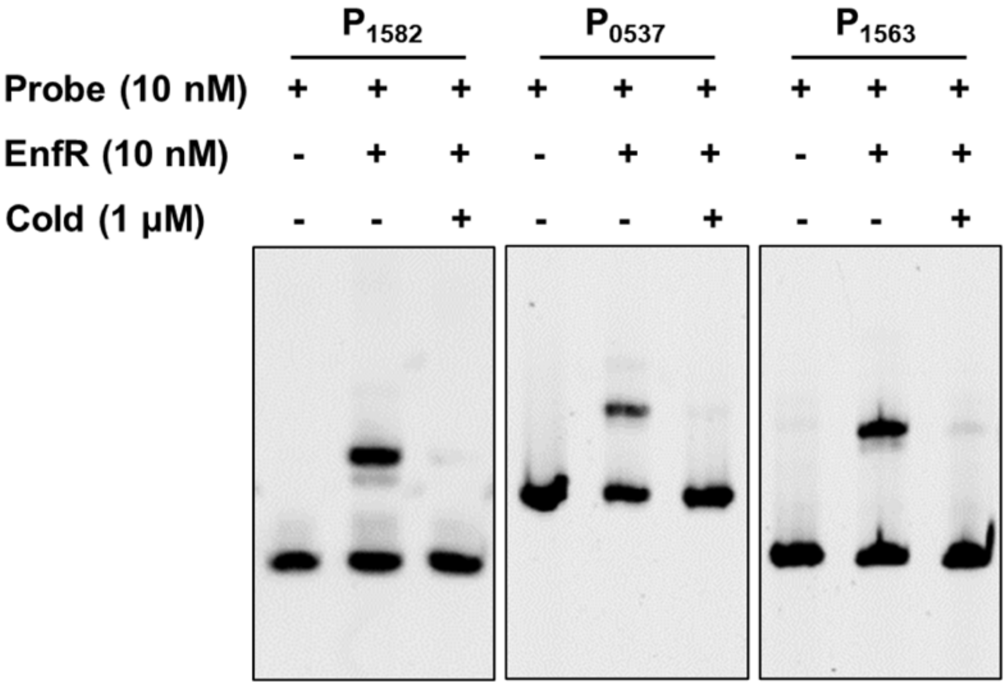

Figure S7. The binding of EnfR to promoters of DEGs. 10 nM EnfR was incubated with the Cy5-labeled promoter fragments (10 nM). P_{1582} and P_{0537} indicate promoter regions of TON_1582 encoding Na+/H+ antiporters and TON_0537 encoding sulfhydrogenase beta subunit SulfI, respectively. P_{1563} represents the promoter region of TON_1565, which encodes formate dehydrogenase. Cold means a specific competitor probe, unlabeled 36-bp target DNA of EnfR.

Table S1. X-ray diffraction data and refinement statistics

Data collection	Wild-type (PDB ID: 8HNO)	T55I (PDB ID: 8HNP)	T55I-L83SeM-C135S
**Data collection**			
Diffraction source	PF-BL17	PF-BL17	PAL-5C
Wavelength (Å)	0.98	0.98	0.98
Rotation range per image (°)	1	0.2	1
Total rotation per image (°)	360	450	360
Exposure time per image (s)	1	1	1
Space group	$P4_32_12$	$P4_32_12$	$P4_32_12$
a, b, c (Å)	102.7,102.7, 90.4	123.2 123.2 82.7	123.7, 123.7, 84.9
Resolution range (Å)	50-2.84	50-3.39	50-3.00
Total no. of reflections	98967	58664	86023
No. of unique reflections	11845	14609	13073
Completeness (%)	98.9 (97.0)	86.9 (85.3)	97.2 (94.8)
Redundancy	8.4 (4.2)	4.02 (3.86)	6.6 (3.9)
I/σ	15.57 (1.88)	16.86 (3.43)	32.76 (5.8)
Rmeas (%)	0.081 (0.469)	0.054 (0.354)	0.076 (0.337)
**Refinement statistics**			
Resolution range (Å)	45.92–2.84	45.84-3.39	35.52-3.00
No. reflections	11824	8115	13691
No. atoms	2219	2127	2278
B-factor	59.2	113.6	69.76
R (Rfree) (%)	22.15 (24.42)	23.57 (27.53)	25.09 (26.74)
**R.M.S. deviations**			
Bonds length	0.003	0.002	0.003
Bond Angles	0.627	0.45	0.662
**Ramachandran plot (%)**			
Most-favored regions	97.05	91.64	88.65
Allowed	2.95	8.36	9.22

Table S2. Primer sequences to amplify the promoter region of the *codh* gene cluster

Primer	Primer Sequence (5'→3')
6-FAM_1017_150_F	GAGAGTTTTACTGTCTCTAAATGAA
1017_150_R	AACCGGAAAAAGCTGGCATTGTTGA

Table S3. Original and substituted sequences of the EnfR-binding site

Mutant	Substituted sequence (5'→3')
Original 36-bp	CCATCTTAAAATCTTTTTGTTTACATTATACGAGGT
A10C-T27G	CCATCTTAACATCTTTTTGTTTACATGATACGAGGT
A11C-T26G	CCATCTTAAACTCTTTTTGTTTACAGTATACGAGGT
T12G-A25C	CCATCTTAAAAGCTTTTTGTTTACCTTATACGAGGT
T14G-A23C	CCATCTTAAAATCGTTTTGTTTCCATTATACGAGGT

Table S4. Primer sequences to amplify the promoter regions of three DEGs

**Primer**	**Primer Sequence (5'→3')**
Cy5_1582_F	AACAGGGTGTTACATAC
1582_R	ATAAATGCCATCTTTCTATC
Cy5_0537/8_F	ACGTATCTCATATTACCACCC
0537/8_R	TTC TGT GCC ATA CCC AC
Cy5_1563_F	AGACTTATTCCTATTTTGTCCTA
1563_R	TTCACTCATTCTCCACAC

Table S5. Strains and plasmids used in this study

Strains/plasmids	Characteristics	Source / Reference
**Strains**		
Escherichia coli DHα	*supE44* Δ*lacU*169 (Φ80 *lacZ* ΔM15) *hsdR17 recA1 endA1 gyrA*96 *thi-1 relAI*	Laboratory collection
**Plasmids**		
pJK1113	pBAD24 with *oriT* of RP4; *nptI*; Ap^r, Km^r	(39)
pJH_EnfRWT	pJK1113 with wild-type TON_1525 gene (*enfR*)	This study
pJH_EnfRDN6	pJK1113 with N-6 region deleted *enfR* gene (Δ1~6 residues)	This study
pJH_EnfRR35A	pJK1113 with $enfR_{R35A}$ gene	This study
pJH_EnfRR43A	pJK1113 with $enfR_{R43A}$ gene	This study
pJH_EnfRR44A	pJK1113 with $enfR_{R44A}$ gene	This study
pJH_EnfRT55I	pJK1113 with $enfR_{T55I}$ gene	This study
pBBR_lux	Broad host range vector containing *luxCDABE* operon; Cm^r	(41)
pJH_road-blocking	Engineered constitutive promoter #1 in pBBR_lux	This study
pJH_steric_hindrance	Engineered constitutive promoter #2 in pBBR_lux	This study
pJH_steric_hindrance_mt	Engineered constitutive promoter #3in pBBR_lux	This study

[a]Ap^r, ampicillin resistant; Km^r, kanamycin resistant, Cm^r, chloramphenicol resistant

Table S6. Sequences of the DNA fragments containing the engineered constitutive promoter with EnfR-binding site

Name	Sequence (5'→3')[a]	Repression mechanism by EnfR binding
Engineered constitutive promoter #1	cGAGCTC**ttgaca**gctagctcagtcctagg**gattgt**gctagcgccgaacacgattccatcttaaaatctttttgtttacattatacgaggtgatctctagagtcgacACTAGTcc	Interfering RNA polymerase proceeding on the promoter
Engineered constitutive promoter #2	cGAGCTCcgtttttatccg**tttacg**cgaacacgattccatct**taaaat**ctttttgtttacattatacgaggtgatcaaca ACTAGTcc	Interfering RNA polymerase binding to the promoter

[a]Nucleotide sequences for restriction enzyme sites are shown in capital letters; -35 and -10 boxes are in bold letters; the EnfR binding sites are underlined.

Table S7. Structural homologs of monomeric EnfR

No	Chain	Z-score	rmsd	Lali	Nres	% id	Description
**1**	3hug-A	8.5	1.8	54	80	13	RNA POLYMERASE SIGMA FACTOR
**2**	7lq4-A	8.5	1.4	53	192	25	RSIG
**3**	6zix-B	8.4	2.0	48	205	31	TRANSCRIPTIONAL REGULATORY PROTEIN RCSB
**4**	3vep-H	8.3	1.9	55	71	25	UNCHARACTERIZED PROTEIN RV3413C/MT3522
**5**	6c03-A	8.0	1.4	49	138	29	PUTATIVE RNA POLYMERASE ECF-SUBFAMILY SIGMA
**6**	3mzy-A	7.9	1.7	58	123	17	RNA POLYMERASE SIGMA-H FACTOR
**7**	3c3w-A	7.9	4.1	55	211	25	TWO-COMPONENT TRANSCRIPTIONAL REGULATORY PROTEIN
**8**	1s7o-C	7.7	7.5	70	108	19	HYPOTHETICAL UPF0122 PROTEIN
**9**	3clo-A	7.5	1.4	48	258	31	TRANSCRIPTIONAL REGULATOR
**10**	6pss-L	7.4	4.1	58	558	22	DNA-DIRECTED RNA POLYMERASE SUBUNIT ALPHA
**11**	1fse-B	7.3	1.4	48	70	23	GERE
**12**	1h0m-B	7.3	1.9	48	230	23	TRANSCRIPTIONAL ACTIVATOR PROTEIN TRAR
**13**	6jcx-F	7.2	3.5	60	186	15	DNA-DIRECTED RNA POLYMERASE SUBUNIT ALPHA
**14**	3qp6-A	7.2	1.6	47	258	19	CVIR TRANSCRIPTIONAL REGULATOR
**15**	6ejq-B	7.0	9.1	69	141	20	TERMINASE SMALL SUBUNIT
**16**	4y13-A	7.0	1.9	48	244	25	TRANSCRIPTIONAL REGULATOR OF FTSQAZ GENE CLUSTER
**17**	3p7n-A	7.0	1.4	47	191	28	SENSOR HISTIDINE KINASE
**18**	6jbq-F	6.9	2.3	55	186	22	DNA-DIRECTED RNA POLYMERASE SUBUNIT ALPHA
**19**	2w48-B	6.9	7.1	64	313	23	SORBITOL OPTERON REGULATOR
**20**	6v7w-E	6.9	1.9	48	234	23	TRANSCRIPTIONAL REGULATOR LASR
**21**	3kln-A	6.8	1.3	46	217	26	TRANSCRIPTIONAL REGULATOR LUXR FAMILY
**22**	5y2v-A	6.7	9.1	63	304	14	RUBISCO OPERON TRANSCRIPTIONAL REGULATOR
**23**	6p18-Q	6.6	4.0	64	156	16	DNA (67-MER) FRAGMENT CARRYING PHAGE-21 PR' PROMO
**24**	2dbb-B	6.6	9.3	64	148	17	PUTATIVE HTH-TYPE TRANSCRIPTIONAL REGULATOR PH006
**25**	7kug-D	6.6	4.4	60	96	20	PROBABLE TRANSCRIPTIONAL REGULATOR WHIB7
**26**	5xt2-B	6.6	1.2	46	204	20	RESPONSE REGULATOR FIXJ
**27**	2ia0-A	6.4	9.6	77	156	19	PUTATIVE HTH-TYPE TRANSCRIPTIONAL REGULATOR PH086
**28**	5hm5-A	6.3	10.8	65	758	18	TOPOISOMERASE V
**29**	2cg4-A	6.2	10.7	72	150	15	REGULATORY PROTEIN ASNC
**30**	6ide-B	6.2	1.8	48	228	23	TRANSCRIPTIONAL REGULATOR RUXR FAMILY

Table S8. Structural homologs of the note-head domain of EnfR

No	Chain	Z-score	rmsd	Lali	Nres	% id	Description
**1**	1i2l-A	6.1	3.2	73	270	8	4-amino-4-deoxychorismate lyase
**2**	2zgi-D	5.4	3.1	69	246	13	Putative 4-amino-4-deoxychorismate lyase
**3**	3qqm-A	4.4	3.1	67	215	18	MLR3007 protein
**4**	5k3w-A	4.1	2.9	71	298	18	CPUTA1
**5**	6kko-A	3.7	3.7	65	170	17	Putative serine phosphatase
**6**	2qby-A	3.7	3.9	63	368	5	Cell division control protein 6 homolog 1
**7**	6m36-I	3.6	3.5	63	104	11	Serine-protein kinase rsbw
**8**	1kt8-B	3.5	3.6	72	366	4	Branched-chain amino acid aminotransferase
**9**	4k6n-A	3.5	3.2	72	352	7	Aminodeoxychorismate lyase
**10**	3ke6-A	3.5	3.3	65	354	18	Protein rv1364c/mt1410
**11**	4psn-C	3.5	2.8	59	221	10	ssDNA binding protein
**12**	4q2d-A	3.3	5.8	58	927	16	CRISPR-associated helicase CAS3
**13**	7ctf-A	3.2	3.8	62	102	8	Origin recognition complex subunit 1
**14**	3ldy-A	3.0	3.3	59	142	12	Restriction endonuclease paci
**15**	3boy-A	2.9	2.9	65	150	14	HutP antitermination complex bound to the HUT mRNA
**16**	3e20-B	2.9	3.8	65	261	8	S.pombe eRF1/eRF3 complex
**17**	1unn-D	2.9	2.3	56	112	9	DNA polymerase III beta subunit
**18**	5a31-N	2.8	3.0	55	703	5	Anaphase-promoting complex subunit 1
**19**	1ldd-A	2.8	2.9	55	74	4	Anaphase-promoting complex
**20**	4psl-C	2.8	3.5	59	148	8	ssDNA binding protein
**21**	3ceb-A	2.8	2.8	64	194	3	A putative 4-amino-4-deoxychorismate lyase (hs_0128)
**22**	6m7z-E	2.7	2.7	49	269	8	Bradyzoite pseudokinase 1
**23**	5iwd-A	2.7	3.0	60	261	7	DNA polymerase processivity factor
**24**	3tek-A	2.6	3.5	61	139	3	ThermoDBP-single stranded DNA binding protein
**25**	4cdb-A	2.6	4.9	58	488	10	Listeriolysin O
**26**	2ols-A	26	5.0	60	725	22	Phosphoenolpyruvate synthase
**27**	1nr3-A	2.6	3.7	58	122	2	DNA binding protein Tfx
**28**	3fds-A	2.5	2.5	54	352	4	DNA polymerase IV
**29**	6em5-D	2.5	3.7	63	107	11	5.8S ribosomal RNA
**30**	3oha-A	2.5	2.7	55	516	2	DNA polymerase eta
